# Interfacial Engineering of Fe_3_O_4_/Biochar via CMC Bridging for Durable Lithium/Sodium-Ion Storage

**DOI:** 10.3390/molecules31203749

**Published:** 2026-10-19

**Authors:** Huijuan Li, Zihan Yang, Chenyi Jia, Zixin Li, Huicong Xia

**Affiliations:** College of Materials Science and Engineering, Zhongyuan Critical Metals Laboratory, Zhengzhou University, Zhengzhou 450001, China

**Keywords:** biochar, Fe_3_O_4_, carboxymethyl cellulose, interfacial engineering, sodium-ion batteries, lithium-ion batteries

## Abstract

The development of cost-effective and durable anode materials derived from renewable biomass is essential for practical lithium- and sodium-ion batteries (LIBs/SIBs). However, conventional strategies for integrating high-capacity metal oxides with biomass carbon often suffer from weak oxide–carbon adhesion, nanoparticle aggregation, and large volume changes during cycling. To overcome these limitations, we present an interfacial engineering approach in which carboxymethyl cellulose (CMC) serves as a molecular bridge to anchor Fe_3_O_4_ nanoparticles in situ onto ginkgo branch-derived biochar (GBC). The abundant hydroxyl and carboxyl groups of CMC simultaneously guide uniform Fe_3_O_4_ nucleation and create a molecular bridge that mediates interfacial chemical interactions that facilitate charge transfer while accommodating volume strain. Structural and compositional analyses confirm the formation of CMC/Fe_3_O_4_/GBC with well-dispersed nanocrystals, a hierarchically porous architecture, and magnetic responsiveness. As an anode, CMC/Fe_3_O_4_/GBC delivers 256 mAh/g at 2000 mA/g in SIBs, substantially higher than that of bare GBC and unmodified Fe_3_O_4_/GBC. Comparable durability is observed in LIBs, where the composite retains 297 mAh/g after 2000 cycles at the same rate. Kinetic studies using cyclic voltammetry, galvanostatic intermittent titration technique, and electrochemical impedance spectroscopy reveal that CMC bridging enhances pseudocapacitive contribution and accelerates ion diffusion. Post-cycling X-ray photoelectron spectroscopy and transmission electron microscopy further confirm the preserved electrode integrity. These results establish a scalable interfacial modification strategy for biomass-derived magnetic composites toward durable alkali-ion storage.

## 1. Introduction

The accelerating deployment of portable electronics, electric vehicles, and grid-scale energy storage systems has driven relentless demand for advanced rechargeable batteries with high energy density, long cycle life, and low cost [1,2,3,4]. While lithium-ion batteries (LIBs) have dominated the market for decades, concerns over the uneven geographic distribution and rising cost of Li resources have motivated intense exploration of sodium-ion batteries (SIBs) as a sustainable complement, particularly for large-scale stationary storage [5,6,7,8]. Nevertheless, the larger ionic radius of Na ion (1.02 Å) compared with Li ion (0.76 Å) leads to sluggish insertion kinetics and severe structural degradation in conventional graphite anodes, making graphite thermodynamically unfavorable for Na ion intercalation [9,10,11]. Consequently, the development of robust and cost-effective anode materials capable of accommodating both Li and Na ion storage remains a pressing challenge.

Biomass-derived biochar provides a renewable and inexpensive porous framework for ion storage, but pristine biochar typically exhibits limited specific capacity and poor rate performance because of insufficient active sites and sluggish ion transport [12]. Introducing high-capacity Fe_3_O_4_ (theoretical capacity: 926 mAh/g) into the carbon matrix is an attractive strategy; however, Fe_3_O_4_ undergoes severe volume expansion (~200%) during repeated ion insertion/extraction, which causes particle pulverization, loss of electrical contact, and rapid capacity decay [13,14,15]. Although combining Fe_3_O_4_ with carbon can buffer volume changes and improve conductivity, conventional physical mixing or simple in situ deposition often results in weak oxide–carbon interfacial adhesion, leading to particle aggregation and detachment during prolonged cycling [16]. Constructing a robust, electronically well-connected interface between Fe_3_O_4_ and biochar is thus essential for durable performance. Carboxymethyl cellulose (CMC), a water-soluble cellulose derivative abundant in hydroxyl and carboxyl groups, can simultaneously chelate metal ions and interact with carbon surfaces, making it a potential bridging agent for constructing robust oxide–carbon interfaces and promoting uniform nanoparticle nucleation [17,18,19]. Nevertheless, the deliberate use of CMC as an interfacial engineering mediator for Fe_3_O_4_/biochar composites, and its impact on Li/Na ion storage kinetics and long-term durability, remains underexplored.

Herein, we employ CMC as a bridging agent to in situ anchor Fe_3_O_4_ nanoparticles onto ginkgo branch-derived biochar (GBC). In a typical procedure, GBC is dispersed in deionized water with FeCl_3_·6H_2_O, followed by the addition of a CMC aqueous solution and ammonia under stirring at 90 °C, which induces the co-precipitation of Fe_3_O_4_ nanoparticles on the biochar surface. The morphology, crystal structure, surface chemical state, and magnetic properties of the resulting CMC/Fe_3_O_4_/GBC composite are systematically characterized by SEM, TEM/HRTEM, HAADF-STEM with EDX mapping, XRD, Raman spectroscopy, TGA, XPS, and VSM, and its electrochemical performance is evaluated in both LIBs and SIBs with emphasis on rate capability and long-term cycling stability. Kinetic analyses via cyclic voltammetry, galvanostatic intermittent titration technique, and electrochemical impedance spectroscopy, combined with post-cycling X-ray photoelectron spectroscopy, reveal that CMC bridging enhances pseudocapacitive contribution, accelerates ion diffusion, and preserves electrode integrity. This work demonstrates a facile and scalable interfacial engineering strategy for biomass-derived magnetic composites toward durable Li/Na-ion storage.

## 2. Results and Discussion

### 2.1. Morphology and Structural Characterization

Surface morphology of the as-prepared samples was initially characterized by scanning electron microscopy (SEM), as presented in Figures S1–S3. Pristine GBC (Figure S1) shows an irregular, fragmented structure with a rough surface and apparent porosity, typical of biomass-derived carbon produced by pyrolysis. Upon Fe_3_O_4_ loading in the absence of CMC (Figure S2), the biochar surface becomes noticeably rougher and displays aggregated particles, suggesting uneven deposition of Fe_3_O_4_. By contrast, CMC/Fe_3_O_4_/GBC (Figure S3) exhibits a more uniform surface texture with finely dispersed particulate features, implying that CMC bridging facilitates homogeneous Fe_3_O_4_ nucleation across the biochar scaffold.

**Figure 1:**
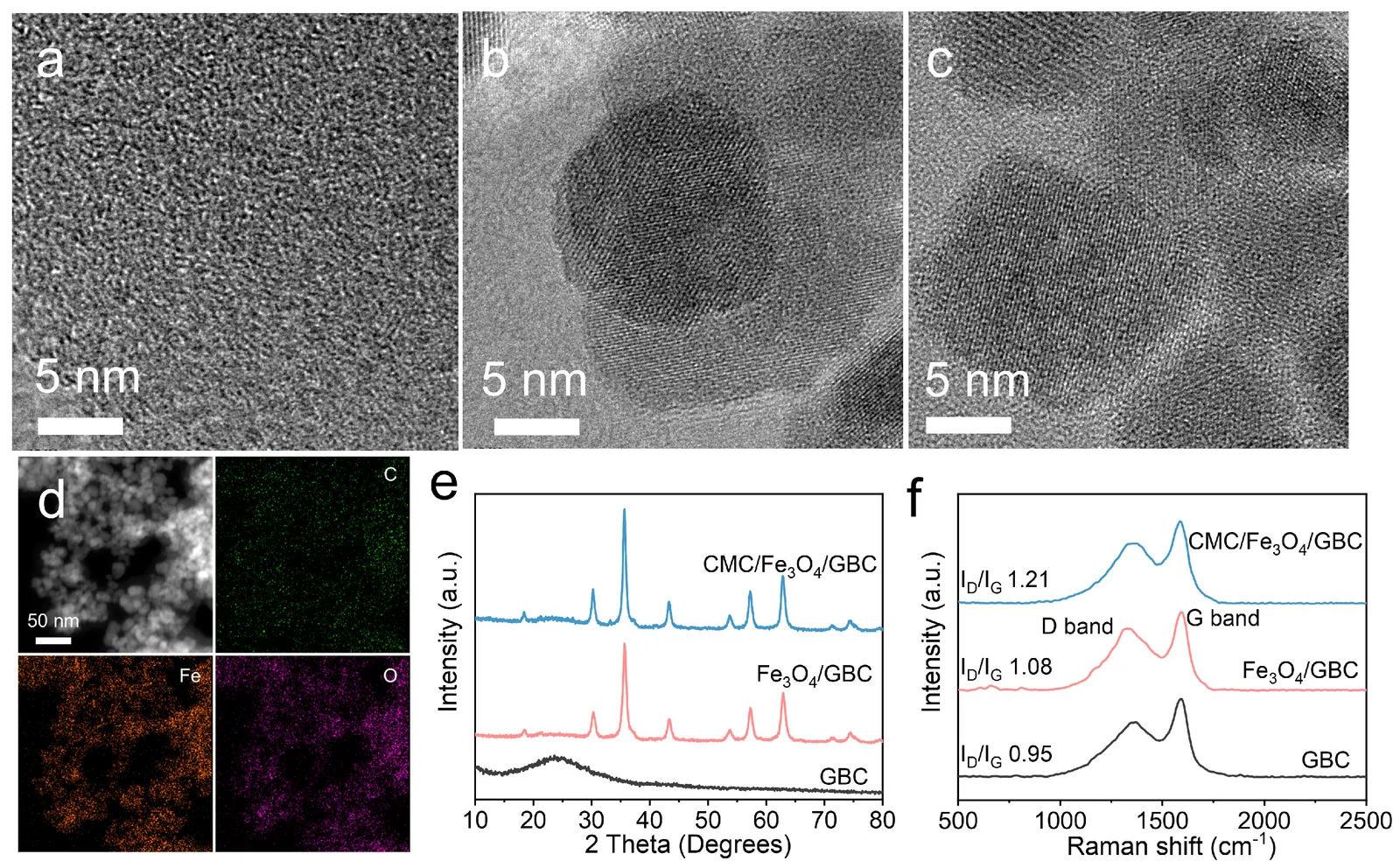
(**a**–**c**) HRTEM micrographs of GBC, Fe_3_O_4_/GBC, and CMC/Fe_3_O_4_/GBC, respectively. (**d**) Corresponding elemental mapping images of the CMC/Fe_3_O_4_/GBC. (**e**) XRD and (**f**) Raman patterns of GBC, Fe_3_O_4_/GBC, and CMC/Fe_3_O_4_/GBC, respectively.

The microstructure was further investigated by transmission electron microscopy (TEM; Figures S4–S6) and high-resolution TEM (HRTEM). As shown in Figure 1a, pristine GBC exhibits a typical amorphous carbon structure without distinguishable lattice fringes, consistent with its low-temperature pyrolysis origin. In contrast, Fe_3_O_4_/GBC (Figure 1b) displays relatively large Fe_3_O_4_ particles (15–20 nm) with clear lattice fringes embedded in the carbon matrix, yet noticeable particle aggregation is evident. Remarkably, CMC/Fe_3_O_4_/GBC (Figure 1c) features substantially smaller and more uniformly dispersed Fe_3_O_4_ nanocrystals (5–10 nm) anchored on the biochar surface, demonstrating that the CMC bridging agent effectively suppresses nanoparticle agglomeration during in situ co-precipitation. The well-resolved lattice fringes with an interplanar spacing of approximately 0.25 nm correspond to the (311) plane of cubic Fe_3_O_4_, confirming its successful crystallization [20].

The elemental distribution in CMC/Fe_3_O_4_/GBC was further verified by HAADF-STEM and energy-dispersive X-ray (EDX) elemental mapping (Figure 1d). The HAADF image reveals bright Fe_3_O_4_ nanoparticles uniformly distributed across the carbon scaffold. The corresponding elemental maps of C, Fe, and O demonstrate homogeneous dispersion of all three elements throughout the composite, with no evidence of localized Fe or O enrichment, corroborating that CMC bridging promotes uniform nucleation and growth of Fe_3_O_4_ on the biochar surface. The EDX spectra (Figure S7) further confirm the presence of C, O, and Fe in both Fe_3_O_4_/GBC and CMC/Fe_3_O_4_/GBC, with the Fe signal appearing only after magnetic modification, consistent with the successful loading of Fe_3_O_4_.

The crystal structures of the samples were characterized by X-ray diffraction (XRD), as presented in Figure 1e. Pristine GBC shows a broad diffraction peak centered at 23^o^, indexed to the (002) plane of amorphous carbon [21]. For Fe_3_O_4_/GBC and CMC/Fe_3_O_4_/GBC, six well-defined diffraction peaks appear at 2*θ* = 30.1°, 35.5°, 43.1°, 53.4°, 57.0°, and 62.6°, which can be respectively assigned to the (220), (311), (400), (422), (511), and (440) planes of cubic magnetite (Fe_3_O_4_, JCPDS No. 19-0629) [22]. No impurity peaks are detected, confirming the phase purity of the as-synthesized Fe_3_O_4_. Notably, the diffraction peaks of CMC/Fe_3_O_4_/GBC are slightly broader and less intense than those of Fe_3_O_4_/GBC, indicating a smaller crystallite size induced by CMC-mediated nucleation, consistent with the HRTEM observations.

Raman spectroscopy was employed to assess the graphitization degree and defect structure of the carbon matrix (Figure 1f). All three samples exhibit two characteristic bands: the D band at approximately 1350 cm^−1^, associated with disordered carbon and structural defects, and the G band at approximately 1580 cm^−1^, corresponding to in-plane stretching of sp^2^-bonded carbon atoms [23]. The intensity ratio of the D band to the G band (I_D_/I_G_) is commonly used to evaluate the degree of graphitization [24]. The intensity ratio of the D band to the G band (I_D_/I_G_) increases from 0.95 for GBC to 1.08 for Fe_3_O_4_/GBC and further to 1.21 for CMC/Fe_3_O_4_/GBC, indicating that the introduction of Fe_3_O_4_ and the CMC bridging layer progressively increases the defect density in the carbon framework. The highest I_D_/I_G_ ratio for CMC/Fe_3_O_4_/GBC suggests the richest defect sites, which can serve as additional active sites for ion adsorption and storage and contribute to the enhanced pseudocapacitive behavior.

Thermogravimetric analysis (TGA) was conducted in air to quantify the composition of the magnetic composites (Figure S8). Both Fe_3_O_4_/GBC and CMC/Fe_3_O_4_/GBC exhibit a gradual weight loss below 200 °C due to the evaporation of adsorbed water, followed by a sharp weight loss between 300 and 500 °C attributed to the combustion of carbonaceous components. The residual weight at 600 °C corresponds to Fe_2_O_3_ formed by the oxidation of Fe_3_O_4_ [25]. CMC/Fe_3_O_4_/GBC shows a residual weight approximately 3.2 wt% higher than that of Fe_3_O_4_/GBC, which is ascribed to the additional carbon content introduced by the CMC bridging layer, confirming the successful incorporation of CMC into the composite.

### 2.2. Surface Chemical State and Magnetic Properties

X-ray photoelectron spectroscopy (XPS) was performed to investigate the surface chemical composition and electronic states of the samples. The survey XPS spectra (Figures S9–S11) confirm the presence of C and O in all three samples, with additional Fe peaks appearing in Fe_3_O_4_/GBC and CMC/Fe_3_O_4_/GBC, consistent with the EDX results. The high-resolution C 1*s* spectra (Figures S9b, S10b and S11b) of all samples can be deconvoluted into C-C/C=C (284.5 eV), C-O (286.0 eV), and O-C=O (288.5 eV) components in CMC/Fe_3_O_4_/GBC relative to Fe_3_O_4_/GBC, indicating enrichment of surface oxygen-containing groups from the CMC layer [26]. Since oxygen species with similar binding energies are also present in Fe_3_O_4_ and the biochar matrix, this observation alone is not taken as direct evidence of interfacial bonding [27]. Instead, the formation of chemical bonding between CMC and Fe_3_O_4_ is supported by the shift in Fe 2*p*^3/2^ binding energy, the shift in the carboxyl stretching mode in FTIR, the retained CMC content revealed by TGA after repeated washing, and the suppressed Fe_3_O_4_ agglomeration observed by TEM.

**Figure 2:**
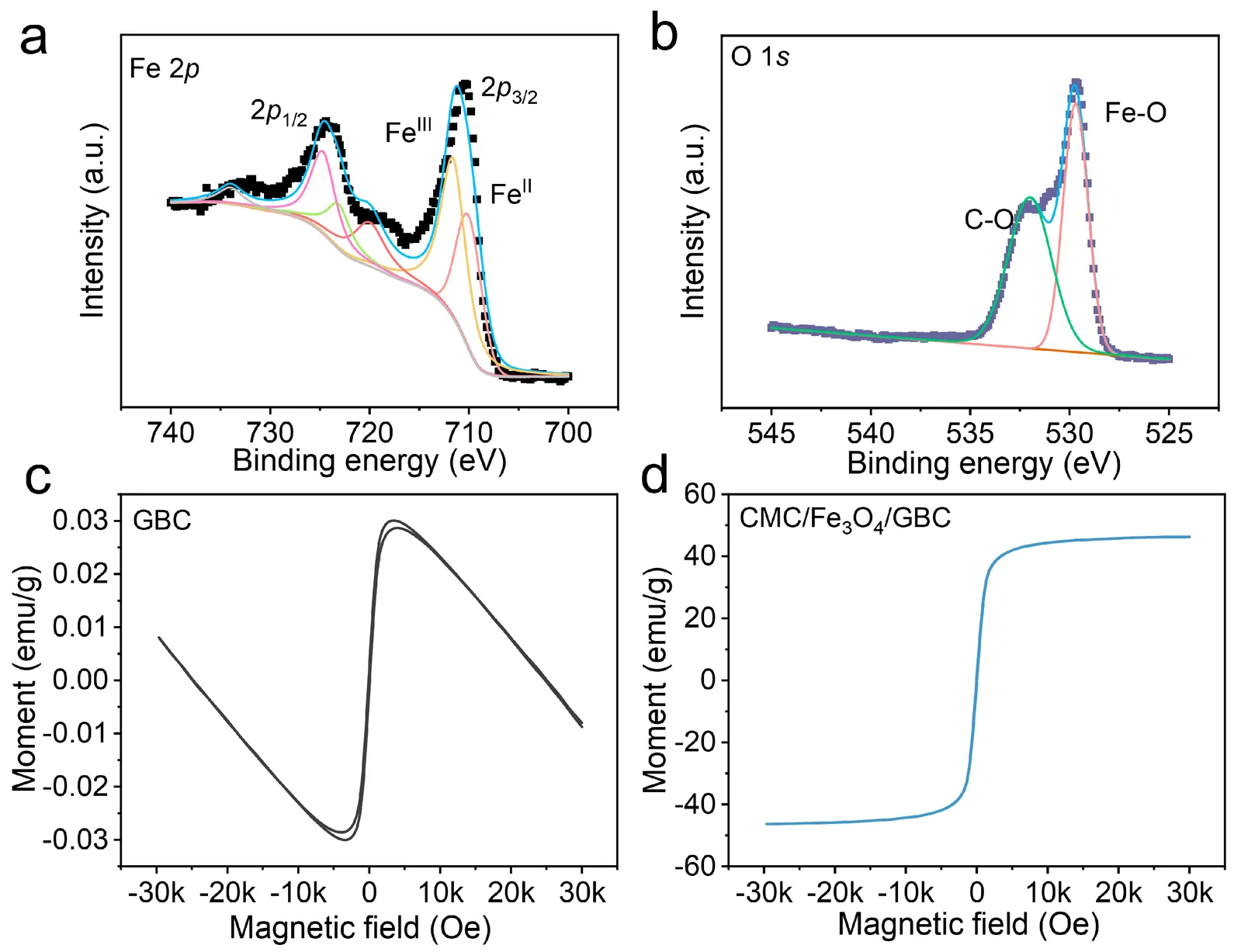
(**a**,**b**) High-resolution XPS spectra of Fe 2*p* and O 1*s* of CMC/Fe_3_O_4_/GBC, respectively. (**c**,**d**) Hysteresis loops of CMC/Fe_3_O_4_/GBC and GBC, respectively.

The high-resolution Fe 2*p* spectrum of CMC/Fe_3_O_4_/GBC (Figure 2a) was fitted with six components, including Fe^2+^ and Fe^3+^ contributions from both 2*p*^3/2^ and 2*p*^1/2^ levels, together with two satellite peaks [28]. The satellite feature at approximately 734 eV is attributed to charge-transfer shake-up processes characteristic of mixed-valence Fe_3_O_4_, further confirming the coexistence of Fe^2+^ and Fe^3+^ in the composite [29]. The Fe 2*p*^3/2^ peak can be deconvoluted into two sub-peaks at 710.2 eV and 712.5 eV, assigned to Fe^2+^ and Fe^3+^ species, respectively [30]. The coexistence of Fe^2+^ and Fe^3+^ is characteristic of magnetite, and the observed satellite peak at 718 eV further confirms the presence of Fe^3+^ [31]. The high-resolution Fe 2*p* spectra were fitted with multiple shake-up satellite components at ~717 eV and ~727 eV. These satellite features are characteristic fingerprints for mixed-valent magnetite (Fe_3_O_4_), and including these components significantly improves the consistency between the fitted envelope and raw experimental spectrum. Reasonable constraints on energy separation and relative intensity ratios among Fe^2+^, Fe^3+^ and satellite peaks were adopted according to standard Fe_3_O_4_ reference spectra. Quantitative analysis of the survey XPS spectra gives a surface Fe:O atomic ratio of approximately 0.75:1 for both Fe_3_O_4_/GBC and CMC/Fe_3_O_4_/GBC, which is close to the theoretical value of 0.75 for stoichiometric Fe_3_O_4_ and distinct from that of Fe_2_O_3_ (0.67) or FeO (1.0). Together with the XRD results and magnetic measurements, this confirms the formation of magnetite in the composite. The high-resolution O 1*s* spectrum (Figure 2b) is fitted into two components: a dominant peak at 530.0 eV attributed to lattice oxygen in Fe-O bonds of Fe_3_O_4_, and a secondary peak at 532.0 eV ascribed to surface oxygen-containing groups (C-O) from the biochar and CMC [32]. The presence of the C-O component suggests strong chemical interaction between the Fe_3_O_4_ surface and the oxygen-rich functional groups of CMC, providing direct evidence for the CMC-mediated interfacial bonding.

The magnetic properties of the samples were evaluated by vibrating sample magnetometry (VSM) at room temperature [33]. As shown in Figure 2c, pristine GBC exhibits a negligible magnetic response with a saturation magnetization (*Ms*) of less than 0.03 emu/g, confirming its nonmagnetic nature. Fe_3_O_4_/GBC (Figure S12) displays a clear ferromagnetic hysteresis loop with a saturation magnetization of approximately 38 emu/g. In contrast, CMC/Fe_3_O_4_/GBC (Figure 2d) displays a typical hysteresis loop with a higher saturation magnetization of approximately 45 emu/g and a small coercivity, indicating superparamagnetic-like behavior at room temperature. The higher *Ms* of CMC/Fe_3_O_4_/GBC compared with Fe_3_O_4_/GBC can be attributed to the smaller, more uniformly dispersed Fe_3_O_4_ nanocrystals with fewer interparticle magnetic interactions. The favorable magnetic responsiveness endows the composite with potential for magnetic separation and recycling, which is advantageous for sustainable electrode manufacturing and post-use recovery.

### 2.3. Lithium-Ion Storage Performance

**Figure 3:**
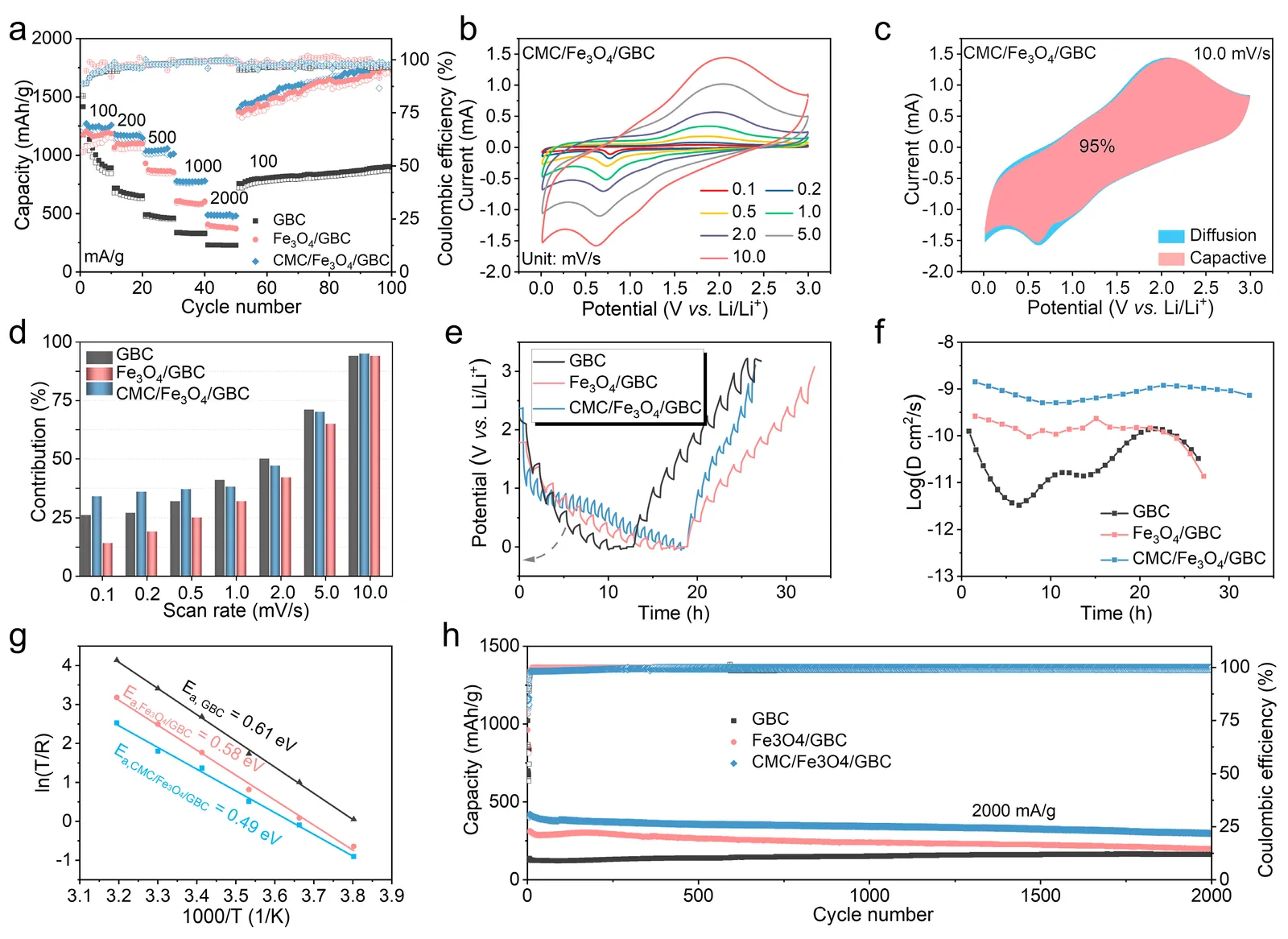
Li ion battery performance. (**a**) Rate performance of GBC, Fe_3_O_4_/GBC, and CMC/Fe_3_O_4_/GBC samples. (**b**) Cyclic voltammetry curves of CMC/Fe_3_O_4_/GBC at various scan rates. (**c**) Capacity contribution ratio of CMC/Fe_3_O_4_/GBC at a scan rate of 10 mV/s. (**d**) Capacity contribution ratios of GBC, Fe_3_O_4_/GBC, and CMC/Fe_3_O_4_/GBC samples at various scan rates. (**e**) GITT profiles of GBC, Fe_3_O_4_/GBC, and CMC/Fe_3_O_4_/GBC samples. (**f**) Li ion diffusion coefficients of GBC, Fe_3_O_4_/GBC, and CMC/Fe_3_O_4_/GBC samples. (**g**) Activation energies of Rct derived from Nyquist plots. (**h**) Long-term cycling performance of GBC, Fe_3_O_4_/GBC, and CMC/Fe_3_O_4_/GBC samples at high current density.

The Li ion storage performance of the three samples was systematically evaluated in CR2032 coin cells. Figure 3a presents the rate capability at current densities ranging from 100 to 2000 mA/g. CMC/Fe_3_O_4_/GBC delivers average reversible capacities of approximately 1200, 1100, 1000, 750, and 500 mAh/g at 100, 200, 500, 1000, and 2000 mA/g, respectively. When the current density is restored to 100 mA/g, a capacity of approximately 1300 mAh/g is recovered, demonstrating excellent rate reversibility. In comparison, Fe_3_O_4_/GBC exhibits capacities of approximately 1200, 1100, 900, 700, and 350 mAh/g at the corresponding current densities, while pristine GBC delivers only 1000, 900, 700, 450, and 200 mAh/g. The substantially superior rate performance of CMC/Fe_3_O_4_/GBC can be attributed to the CMC-bridged interface, which facilitates rapid electron transfer and ion diffusion while maintaining structural integrity under high current stress.

Cyclic voltammetry (CV) was conducted at scan rates from 0.1 to 10 mV/s to elucidate the Li ion storage kinetics. The CV curves of CMC/Fe_3_O_4_/GBC (Figure 3b) display a pair of broad redox peaks centered at approximately 0.8 V (cathodic) and 1.5 V (anodic), corresponding to the reversible conversion reaction between Fe_3_O_4_ and Li ions. As the scan rate increases, the peak currents grow proportionally without significant peak shift, indicating fast reaction kinetics and low polarization. Figure S13a–c displays the representative discharge–charge profiles of GBC, Fe_3_O_4_/GBC, and CMC/Fe_3_O_4_/GBC electrodes at a current density of 100 mA/g, which align with the shape of CV curves. The voltage profiles are characteristic of Fe_3_O_4_-based materials. For comparison, the CV curves of GBC and Fe_3_O_4_/GBC at various scan rates are provided in Figures S14–S16, respectively, both showing less-defined redox features and lower current responses than CMC/Fe_3_O_4_/GBC. To quantify the capacitive and diffusion-controlled contributions, the current response at each scan rate was analyzed according to the power-law relationship i = av^b^ [34]. At a scan rate of 10 mV/s (Figure 3c), the capacitive contribution reaches as high as 95%, with only 5% attributed to diffusion-controlled processes. Figure 3d further illustrates that the capacitive contribution ratio increases progressively with scan rate for all three samples; notably, CMC/Fe_3_O_4_/GBC consistently exhibits the highest capacitive contribution across all scan rates, rising from approximately 32% at 0.1 mV/s to 95% at 10 mV/s. The detailed capacitive contribution analyses for GBC and Fe_3_O_4_/GBC are presented in Figures S14 and S15, respectively, confirming the trend observed in Figure 3d. This dominant pseudocapacitive behavior, facilitated by the abundant interfacial sites and short ion diffusion paths in the CMC-bridged nanostructure, accounts for the exceptional rate capability.

The Li ion diffusion kinetics were further investigated by galvanostatic intermittent titration technique (GITT). As shown in Figure 3e, CMC/Fe_3_O_4_/GBC exhibits the smallest voltage hysteresis during the pulse-relaxation process, while pristine GBC shows the largest overpotential. The calculated Li ion diffusion coefficients (D_Li_) are plotted in Figure 3f. CMC/Fe_3_O_4_/GBC maintains D_Li_ values in the range of 10^−8^ to 10^−9^ cm^2^/s, which is approximately one to two orders of magnitude higher than those of Fe_3_O_4_/GBC (10^−9^ to 10^−11^ cm^2^/s) and GBC (10^−10^ to 10^−12^ cm^2^/s). The accelerated ion diffusion in CMC/Fe_3_O_4_/GBC further confirms that the CMC-bridged interface constructs efficient ion transport pathways.

The charge-transfer resistance and its temperature dependence were analyzed by electrochemical impedance spectroscopy (EIS) at various temperatures (Figures S14i–S16i). The activation energy (E_a_) for charge transfer was derived from the Arrhenius relationship, as shown in Figure 3g. CMC/Fe_3_O_4_/GBC exhibits the lowest charge-transfer resistance at all measured temperatures, reflected by the highest ln(T/R) values. Although the apparent activation energy of CMC/Fe_3_O_4_/GBC is slightly higher than that of the other samples, its absolute charge-transfer resistance remains substantially lower across the entire temperature range, indicating that the CMC-bridged interface provides intrinsically favorable electron-conducting pathways that dominate the overall charge-transfer process.

The long-term cycling stability was evaluated at a high current density of 2000 mA/g (Figure 3h). After an initial activation process, CMC/Fe_3_O_4_/GBC maintains a stable reversible capacity of approximately 297 mAh/g over 2000 cycles with nearly 100% Coulombic efficiency. In contrast, Fe_3_O_4_/GBC retains approximately 250 mAh/g with gradual capacity fading, and pristine GBC delivers only approximately 150 mAh/g. The remarkable long-term durability of CMC/Fe_3_O_4_/GBC underscores the effectiveness of CMC bridging in buffering the volume expansion of Fe_3_O_4_ and preserving electrode integrity during prolonged Li ion insertion/extraction.

### 2.4. Sodium-Ion Storage Performance

**Figure 4:**
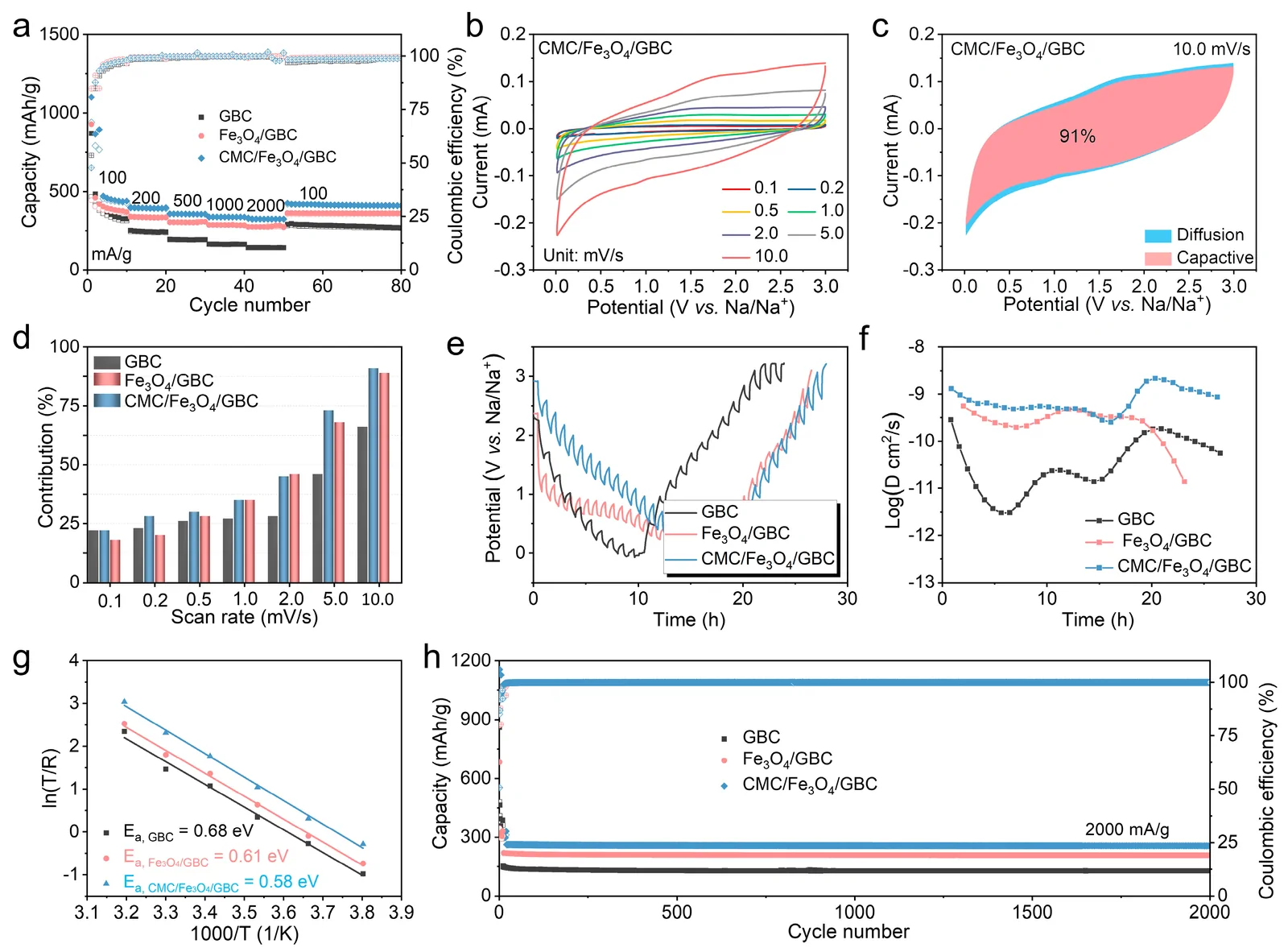
Na ion battery performance. (**a**) Rate performance of GBC, Fe_3_O_4_/GBC, and CMC/Fe_3_O_4_/GBC samples. (**b**) Cyclic voltammetry curves of CMC/Fe_3_O_4_/GBC at various scan rates. (**c**) Capacity contribution ratio of CMC/Fe_3_O_4_/GBC at a scan rate of 10 mV/s. (**d**) Capacity contribution ratios of GBC, Fe_3_O_4_/GBC, and CMC/Fe_3_O_4_/GBC samples at various scan rates. (**e**) GITT profiles of GBC, Fe_3_O_4_/GBC, and CMC/Fe_3_O_4_/GBC samples. (**f**) Na ion diffusion coefficients of GBC, Fe_3_O_4_/GBC, and CMC/Fe_3_O_4_/GBC samples. (**g**) Activation energies of Rct derived from Nyquist plots. (**h**) Long-term cycling performance of GBC, Fe_3_O_4_/GBC, and CMC/Fe_3_O_4_/GBC samples at high current density.

Encouraged by the superior Li ion storage performance, the Na ion storage capability of the samples was similarly evaluated. Figure 4a shows the rate performance at current densities from 100 to 2000 mA/g. CMC/Fe_3_O_4_/GBC delivers reversible capacities of approximately 500, 470, 450, 400, and 350 mAh/g at 100, 200, 500, 1000, and 2000 mA/g, respectively, and recovers to approximately 450 mAh/g when the current returns to 100 mA/g. Fe_3_O_4_/GBC and GBC exhibit markedly lower capacities across all current densities, with GBC showing the poorest rate retention. The enhanced Na ion storage of CMC/Fe_3_O_4_/GBC is particularly noteworthy given the larger ionic radius of Na ion, which typically imposes more stringent requirements on electrode structure and kinetics.

The CV curves of CMC/Fe_3_O_4_/GBC at scan rates from 0.1 to 10 mV/s (Figure 4b) display a quasi-rectangular shape with broad redox features, indicating that capacitive Na-ion storage dominates. Discharge–charge profiles of GBC, Fe_3_O_4_/GBC, and CMC/Fe_3_O_4_/GBC electrodes at 100 mA/g are shown in Figure S13d–f, and their shapes agree well with the corresponding CV curves. For comparison, the CV curves of GBC and Fe_3_O_4_/GBC in SIBs are provided in Figures S17–S19; both exhibit narrower current envelopes and less favorable kinetics than CMC/Fe_3_O_4_/GBC. The capacitive contribution analysis (Figure 4c) reveals that 91% of the total capacity is capacitive at 10 mV/s, with only 9% from diffusion-controlled processes. As summarized in Figure 4d, the capacitive contribution of CMC/Fe_3_O_4_/GBC increases from approximately 23% at 0.1 mV/s to 92% at 10 mV/s, consistently outperforming the other two samples. The detailed capacitive contribution analyses for GBC and Fe_3_O_4_/GBC in SIBs are presented in Figures S17 and S18, respectively. The high pseudocapacitive contribution is especially beneficial for Na ion storage, as it circumvents the slow solid-state diffusion of the larger Na ions.

GITT measurements (Figure 4e) confirm that CMC/Fe_3_O_4_/GBC exhibits the smallest voltage polarization among the three samples. In the GITT profiles, the change in potential behavior at approximately 10–20 h corresponds to the conversion reaction region of Fe_3_O_4_, where the electrode undergoes the transformation of Fe_3_O_4_ into metallic Fe and Li_2_O/Na_2_O, accompanied by SEI formation. This region is kinetically sluggish and exhibits the most pronounced voltage hysteresis. CMC/Fe_3_O_4_/GBC shows the smallest hysteresis in this window, indicating that the CMC-bridged interface effectively facilitates charge transfer and ion diffusion during the conversion step, consistent with its superior rate capability and long-term cycling stability. The calculated Na ion diffusion coefficients (Figure 4f) range from 10^−9^ to 10^−10^ cm^2^/s for CMC/Fe_3_O_4_/GBC, which is one to two orders of magnitude higher than those of Fe_3_O_4_/GBC and GBC. The diffusion coefficients derived from GITT exhibit a non-monotonic variation with the state of discharge, which is characteristic of conversion-type anodes. The initial decrease is associated with ion accumulation and increasing diffusion barriers, the partial recovery reflects the onset of the conversion reaction and the formation of nanoscale Fe^0^ domains that facilitate charge transfer, and the final decrease arises from SEI growth and the accumulation of insulating Li_2_O/Na_2_O species. CMC/Fe_3_O_4_/GBC exhibits the smallest fluctuation and the highest diffusion coefficient throughout the process, confirming that the CMC-bridged interface stabilizes the ion transport kinetics and mitigates the polarization associated with the conversion reaction.

The temperature-dependent EIS analysis (Figures S17i–S19i; Figure 4g) reveals a similar trend to that observed in LIBs, with CMC/Fe_3_O_4_/GBC exhibiting the lowest charge-transfer resistance at all temperatures. The long-term cycling test at 2000 mA/g (Figure 4h) demonstrates that CMC/Fe_3_O_4_/GBC maintains a stable capacity of approximately 256 mAh/g over 2000 cycles with nearly 100% Coulombic efficiency, significantly outperforming Fe_3_O_4_/GBC (208 mAh/g) and GBC (128 mAh/g). These results collectively demonstrate that the CMC-mediated interfacial engineering strategy is equally effective for Na ion storage, enabling durable high-rate performance in both LIBs and SIBs.

### 2.5. Post-Cycling Electrode Analysis

**Figure 5:**
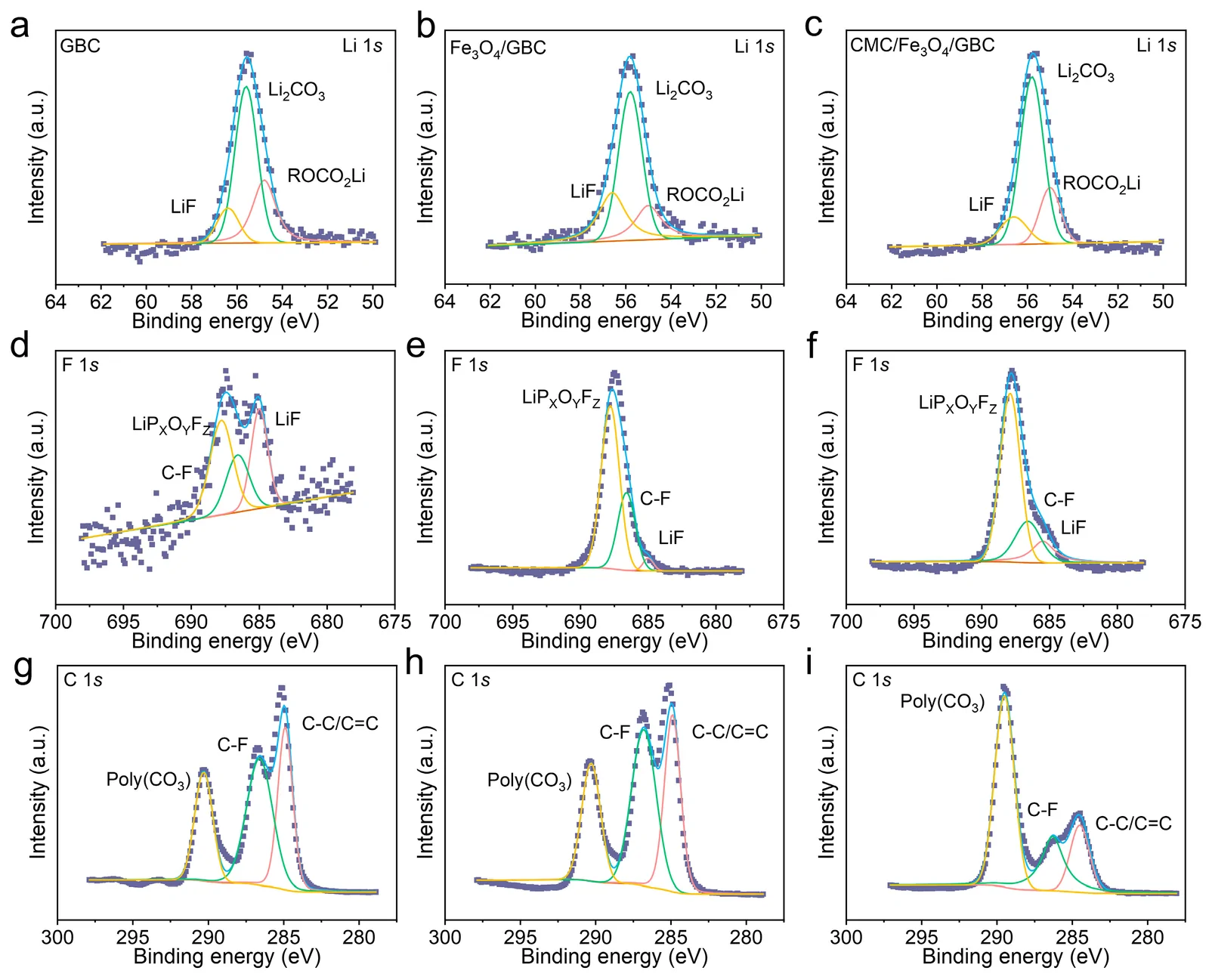
(**a**,**d**,**g**) High-resolution Li 1*s*, F 1*s*, and C 1*s* XPS spectra of GBC after cycling in LIBs; (**b**,**e**,**h**) High-resolution Li 1*s*, F 1*s*, and C 1*s* XPS spectra of Fe_3_O_4_/GBC after cycling in LIBs; (**c**,**f**,**i**) High-resolution Li 1*s*, F 1*s*, and C 1*s* XPS spectra of CMC/Fe_3_O_4_/GBC after cycling in LIBs.

To gain deeper mechanistic insight into the superior cycling durability of CMC/Fe_3_O_4_/GBC, post-cycling XPS was performed on all three electrodes after rate capability test cycling in LIBs (Figures S20–S22). The Li 1*s* spectra of all cycled electrodes can be deconvoluted into three distinct components centered at approximately 56.0, 55.0, and 54.0 eV, which are respectively assigned to lithium fluoride (LiF), lithium carbonate (Li_2_CO_3_), and lithium alkyl carbonate (ROCO_2_Li), all of which are well-recognized constituents of the solid-electrolyte interphase (SEI) layer formed on anode surfaces [35]. A close comparison among the three samples reveals a clear trend in SEI composition. For pristine GBC (Figure 5a), the Li 1*s* spectrum exhibits a relatively broad Li_2_CO_3_ peak accompanied by a substantial ROCO_2_Li component, and the overall spectrum is noticeably noisy with significant scatter in the raw data points, suggesting the formation of a thick, chemically heterogeneous SEI layer. In contrast, the Li 1*s* spectrum of CMC/Fe_3_O_4_/GBC (Figure 5c) displays a markedly sharper and more intense Li_2_CO_3_ peak with a conspicuously diminished ROCO_2_Li fraction, while the Fe_3_O_4_/GBC electrode (Figure 5b) shows an intermediate behavior. The reduced organic ROCO_2_Li content and enhanced inorganic Li_2_CO_3_ proportion in CMC/Fe_3_O_4_/GBC collectively indicate that the CMC-bridged interface promotes the formation of a more inorganic-dominated SEI, which is generally regarded as mechanically more robust and ionically more conductive than an organic-rich SEI.

The F 1*s* spectra provide further corroborating evidence for the SEI composition differences among the three electrodes. All spectra are fitted into three components: LiP_x_O_y_F_z_ at approximately 687.0 eV (originating from the decomposition of LiPF_6_ salt), C-F at approximately 688.0 eV (arising from PVDF binder residues), and LiF at approximately 685.0 eV (a key inorganic SEI component) [36]. The F 1*s* spectrum of GBC (Figure 5d) is characterized by extensive noise and poorly resolved peak features, with a high background level that is consistent with a thick, irregular SEI layer containing a mixture of decomposition products. In comparison, the Fe_3_O_4_/GBC spectrum (Figure 5e) shows improved peak definition with a dominant LiP_x_O_y_F_z_ component. Remarkably, the CMC/Fe_3_O_4_/GBC electrode (Figure 5f) exhibits the cleanest and best-resolved F 1*s* spectrum, featuring a sharp, dominant LiP_x_O_y_F_z_ peak accompanied by well-defined C-F and LiF sub-peaks. The high spectral quality and clear peak separation for CMC/Fe_3_O_4_/GBC are consistent with the formation of a thinner, more uniform, and chemically homogeneous SEI layer, which minimizes XPS signal attenuation from an excessively thick interphase.

The most striking difference among the three cycled electrodes is revealed by the high-resolution C 1*s* spectra, which are deconvoluted into polycarbonate (Poly(CO_3_), ~290.0 eV), C-F (~287.0 eV), and C-C/C=C (~284.5 eV) components [37]. For both GBC (Figure 5g) and Fe_3_O_4_/GBC (Figure 5h), the C 1*s* spectra are dominated by the C-C/C=C peak, which primarily reflects the underlying carbonaceous biochar substrate, with secondary Poly(CO_3_) and C-F contributions from the SEI overlayer. This dominance of the substrate signal indicates that the SEI formed on these two electrodes is either relatively thin or porous, allowing substantial XPS probing of the underlying carbon. In stark contrast, the C 1*s* spectrum of CMC/Fe_3_O_4_/GBC (Figure 5i) is overwhelmingly dominated by the inorganic Poly(CO_3_) peak, while the C-C/C=C substrate signal is significantly attenuated. This pronounced inversion of peak intensities, where the SEI-derived Poly(CO_3_) component surpasses the substrate C-C/C=C signal, provides compelling evidence that CMC/Fe_3_O_4_/GBC is covered by a dense, continuous, and inorganic-rich SEI layer that effectively shields the underlying carbon substrate from XPS detection. The abundant Poly(CO_3_) and LiF components in the SEI of CMC/Fe_3_O_4_/GBC are known to confer high mechanical strength and excellent Li ion conductivity, enabling the SEI to accommodate the volume strain of Fe_3_O_4_ during repeated lithiation/delithiation while maintaining structural integrity. Furthermore, the inorganic-rich SEI effectively passivates the electrode surface, suppressing continuous parasitic electrolyte decomposition and preventing the growth of a thick, resistive interphase over prolonged cycling. 

Post-cycling TEM images of CMC/Fe_3_O_4_/GBC at different magnifications after rate capability test in LIBs are shown in Figure S23. The low-magnification image (Figure S23a) reveals that the biochar scaffold remains structurally intact without pulverization or fragmentation. At intermediate magnification (Figure S23b), Fe_3_O_4_ nanocrystals retain their small size (~5–10 nm) and uniform dispersion on the carbon support, with no significant agglomeration or particle coalescence. The high-resolution image (Figure S23c) confirms well-resolved lattice fringes of ~0.25 nm corresponding to the (311) plane of Fe_3_O_4_, and the nanocrystals remain intimately embedded in the amorphous carbon matrix with no interfacial gaps. A thin conformal SEI layer is also visible on particle surfaces. Collectively, the post-cycling XPS and TEM results establish that CMC-mediated interfacial engineering not only preserves the nanostructural integrity of the electrode but also directs the formation of a favorable, inorganic-rich SEI layer, both of which are indispensable for the exceptional long-term cycling stability observed for CMC/Fe_3_O_4_/GBC in both LIBs and SIBs.

**Figure 6:**
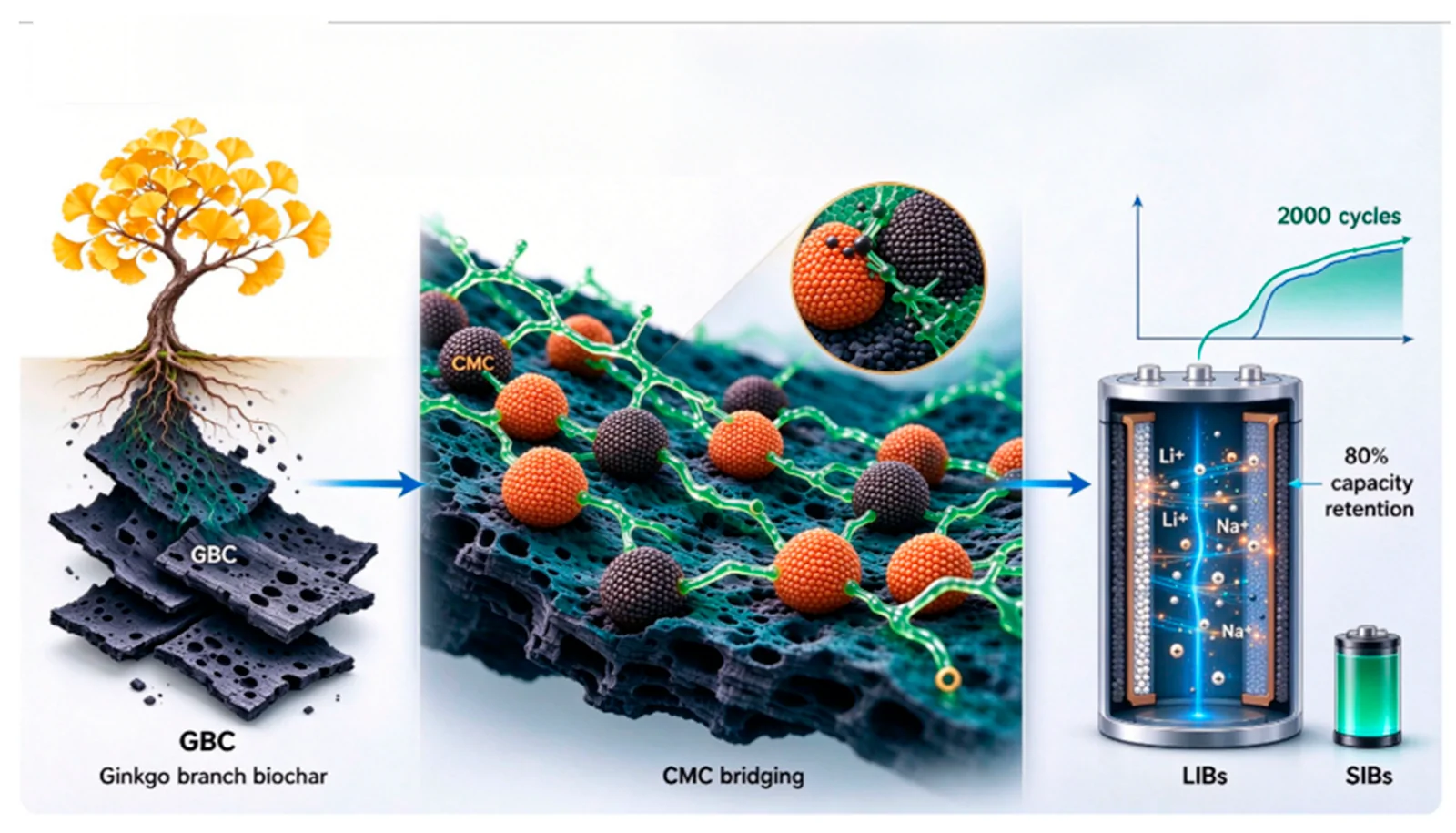
Schematic illustration for the construction of CMC/Fe_3_O_4_/GBC anode.

Overall, the comprehensive characterization and electrochemical analyses demonstrate that CMC-mediated interfacial engineering plays a multifaceted role (Figure 6): it (i) promotes uniform dispersion of Fe_3_O_4_ nanocrystals, (ii) constructs a robust oxide–carbon interface that facilitates charge transfer and ion diffusion, (iii) enhances pseudocapacitive contribution for high-rate capability, (iv) buffers volume expansion during cycling, and (v) promotes the formation of a stable, inorganic-rich SEI layer. These synergistic effects collectively enable CMC/Fe_3_O_4_/GBC to deliver outstanding rate performance and long-term durability in both Li and Na ion batteries.

## 3. Experimental Section

### 3.1. Materials

Ferric chloride hexahydrate (FeCl_3_·6H_2_O) was purchased from Beijing InnoChem Science & Technology Co., Ltd. (Beijing, China). Carboxymethyl cellulose (CMC), hydrochloric acid (HCl), and ammonia solution were supplied by Sinopharm Chemical Reagent Co., Ltd. (Shanghai, China). Ginkgo branches were collected from local natural sources. Battery-grade electrolyte was obtained from Dodo Chem Co., Ltd. (Suzhou, China), and copper foil (Cu foil) was sourced from Canrd Technology Co., Ltd. (Shenzhen, China) All chemical reagents were used as-received without further purification. Deionized (DI) water (18.2 MΩ·cm) produced by a Millipore ultrapure water system (Merck KGaA, Darmstadt, Germany) was utilized throughout all experimental procedures.

### 3.2. Synthesis 

Preparation of Ginkgo Branch Biochar (GBC). Ginkgo branch samples were thoroughly rinsed with deionized water and air-dried at room temperature, then further dried in an oven at 70 °C. The dried branches were ground into a fine powder and sieved through a 60-mesh screen to yield a uniform precursor. An appropriate amount of the precursor powder was placed in a quartz boat and pyrolyzed in a tube furnace at 500 °C for 3 h under a continuous nitrogen flow to maintain an oxygen-free atmosphere. After cooling to room temperature, the resulting biochar was washed with 1 mol/L hydrochloric acid to remove inorganic impurities formed during carbonization, followed by repeated rinsing with deionized water (3–5 times) until the filtrate was neutral. The purified biochar was dried at 70 °C, weighed, and stored in a sealed glass vial for subsequent use.

Preparation of Fe_3_O_4_/GBC and CMC/Fe_3_O_4_/GBC. Typically, 0.5 g of as-prepared GBC was dispersed in a beaker containing an appropriate volume of deionized water, followed by the addition of 2.1 g of FeCl_3_·6H_2_O. The mixture was stirred until all reagents were fully dissolved. The beaker was then transferred to a water bath preheated to 90 °C. After adding 10 mL of a 1 wt% carboxymethyl cellulose (CMC) aqueous solution, 10 mL of ammonia solution was rapidly introduced under continuous stirring. The reaction was maintained for 30 min, after which heating was discontinued and the mixture was allowed to cool to room temperature. The product was repeatedly washed with deionized water until the filtrate reached neutral pH and dried at 60 °C to afford the CMC-modified magnetic composite (CMC/Fe_3_O_4_/GBC). For comparison, the unmodified magnetic biochar (Fe_3_O_4_/GBC) was prepared via an identical procedure except that the CMC solution was omitted.

### 3.3. Electrochemical Characterization

Electrochemical measurements were performed using CR2032 coin-type cells (Canrd Technology Co., Ltd., Shenzhen, China). The working electrodes were prepared by mixing the active material, carbon black, and poly(vinylidene fluoride) (PVDF) at a mass ratio of 7:2:1 in N-methyl-2-pyrrolidone (NMP). The slurry was obtained by ball milling at 200 rpm for 8 h, then coated onto copper foil (Canrd Technology Co., Ltd., Shenzhen, China) and dried at 60 °C for 24 h. The mass loading of the active material was 0.4–0.6 mg/cm^2^. Sodium foil was used as the counter electrode, and 1.0 M NaPF_6_ dissolved in a mixed solvent of ethylene carbonate and diethyl carbonate (EC/DEC, 1:1 *v*/*v*; Dodo Chem Co., Ltd., Suzhou, China) served as the electrolyte. A Whatman GF/D glass fiber membrane was used as the separator. Cells were assembled in an Ar-filled glovebox (MBraun MB-Labstar Pro, M. Braun Inertgas-Systeme GmbH, Garching, Germany) with H_2_O and O_2_ concentrations below 0.5 ppm. Galvanostatic discharge/charge tests were conducted on a CT-3008 battery testing system (Shenzhen Neware Co., Ltd., Shenzhen, China) at 30 °C within a voltage window of 0.01–3.0 V at various current densities. Cyclic voltammetry (CV) and electrochemical impedance spectroscopy (EIS) were performed on a CHI 660E/760E electrochemical workstation (CH Instruments, Inc., Austin, TX, USA). Galvanostatic intermittent titration technique (GITT) measurements were carried out by applying a current pulse of 100 mA/g for 30 min, followed by a 30-min relaxation period.

### 3.4. Physical Characterization

The morphology of the samples was examined by transmission electron microscopy (TEM; FEI Tecnai G2 F30 Thermo Fisher Scientific, Hillsboro, OR, USA; Hitachi HT7700, Hitachi High-Technologies Corporation, Tokyo, Japan) and scanning electron microscopy (SEM; Hitachi S-5500, Hitachi High-Technologies Corporation, Tokyo, Japan). High-angle annular dark-field scanning TEM (HAADF-STEM) and energy-dispersive X-ray (EDX) elemental mapping were acquired on a Philips FEI Tecnai 20 operated at an accelerating voltage of 200 kV. X-ray diffraction (XRD) patterns were recorded on a Rigaku D/Max 2500/PC diffractometer (Rigaku Corporation, Tokyo, Japan) using Cu Kα radiation (λ = 1.5406 Å) at 40 kV and 30 mA. Thermogravimetric analysis (TGA) was performed on a PerkinElmer Diamond TG/DTA instrument (PerkinElmer, Inc., Waltham, MA, USA) from 30 to 800 °C at a heating rate of 5 °C/min in air. X-ray photoelectron spectroscopy (XPS) was conducted on a Kratos Axis Ultra DLD spectrometer (Kratos Analytical Ltd., Manchester, UK) using monochromated Al Kα radiation (hν = 1486.6 eV) at 15 kV with a pass energy of 160 eV. Raman spectra were collected on a Horiba Jobin Yvon LabRAM HR spectrometer (Horiba Ltd., Kyoto, Japan) equipped with a 532 nm excitation laser, a 600 gr/mm diffraction grating, and a 50× objective (N.A. = 0.50). The laser power was set to 1 mW at the sample surface to avoid laser-induced degradation. Each spectrum was accumulated over 3 cycles with an acquisition time of 10 s per cycle, and the reported spectra represent the average of these accumulations. Magnetic properties were measured at room temperature using a vibrating sample magnetometer (VSM; Lake Shore 7404, Horiba Ltd., Kyoto, Japan) to obtain M-H hysteresis loops.

## 4. Conclusions

In summary, we have demonstrated a facile interfacial engineering strategy that employs carboxymethyl cellulose (CMC) as a bridging agent to in situ anchor Fe_3_O_4_ nanoparticles onto ginkgo branch-derived biochar (GBC). The CMC molecules, rich in hydroxyl and carboxyl groups, simultaneously chelate iron ions and interact with the carbon surface, promoting uniform nucleation of Fe_3_O_4_ nanocrystals and constructing a robust oxide–carbon interface. Systematic characterization confirms that CMC/Fe_3_O_4_/GBC features well-dispersed Fe_3_O_4_ nanocrystals, abundant porous structure, and favorable magnetic responsiveness. Electrochemical evaluations reveal that the CMC-bridged composite delivers exceptional rate capability and long-term cycling stability in both Li and Na ion batteries, outperforming both pristine GBC and unmodified Fe_3_O_4_/GBC. Kinetic analyses demonstrate that CMC bridging enhances pseudocapacitive contribution, accelerates ion diffusion, and lowers charge-transfer resistance. Post-cycling XPS and TEM analyses further verify that the CMC-mediated interface promotes the formation of a stable, inorganic-rich solid-electrolyte interphase and preserves electrode integrity during prolonged cycling. This work offers a scalable and sustainable approach for interfacial engineering of biomass-derived magnetic composites toward durable alkali-metal-ion storage, and the CMC-bridging strategy can be readily extended to other metal oxide/carbon composite systems for advanced energy storage applications.

## Data Availability

The data presented in this study are available on request from the corresponding author.

## Supplementary Materials

The following supporting information can be downloaded at: https://www.mdpi.com/article/10.3390/molecules31203749/s1.
