# Supplementary material for: Interfacial Engineering of Fe3O4/Biochar via CMC Bridging for Durable Lithium/Sodium-Ion Storage
Source: Molecules. 2026 Oct 19;31(20):3749. doi: 10.3390/molecules31203749 (PMC13707597; doi:10.3390/molecules31203749)
Supplement: Supplementary file 1 [file molecules-31-03749-s001.zip › molecules-4614998-supplementary.pdf]

Supporting information

Interfacial engineering of Fe_3O_4/biochar via CMC bridging for durable lithium/sodium-ion storage

Huijuan Li, Zihan Yang, Chenyi Jia, Zixin Li and Huicong Xia *

College of Materials Science and Engineering, Zhongyuan Critical Metals Laboratory, Zhengzhou University, Zhengzhou 450001, China
E-mail: hcxia9209@zzu.edu.cn

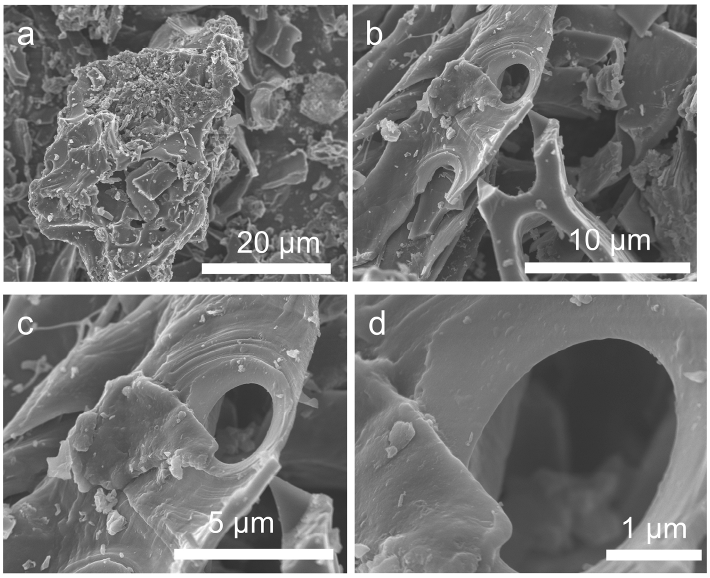

Figure S1. (a-d) SEM images of GBC at different magnifications.

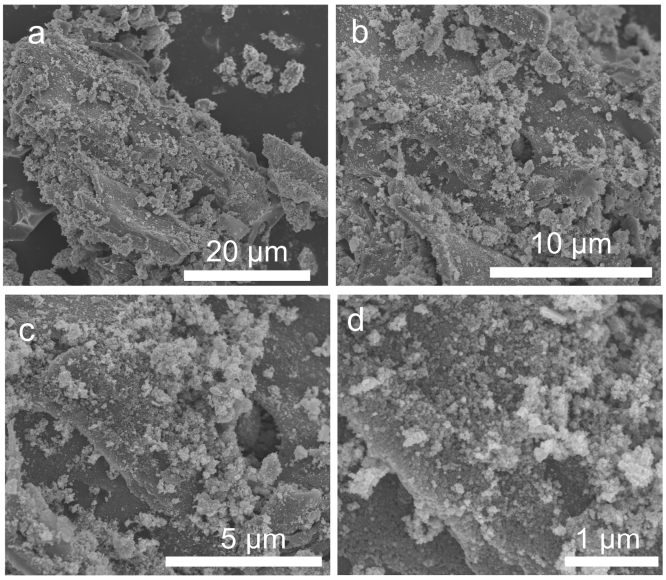

Figure S2. (a-d) SEM images of Fe_3O_4/GBC at different magnifications.

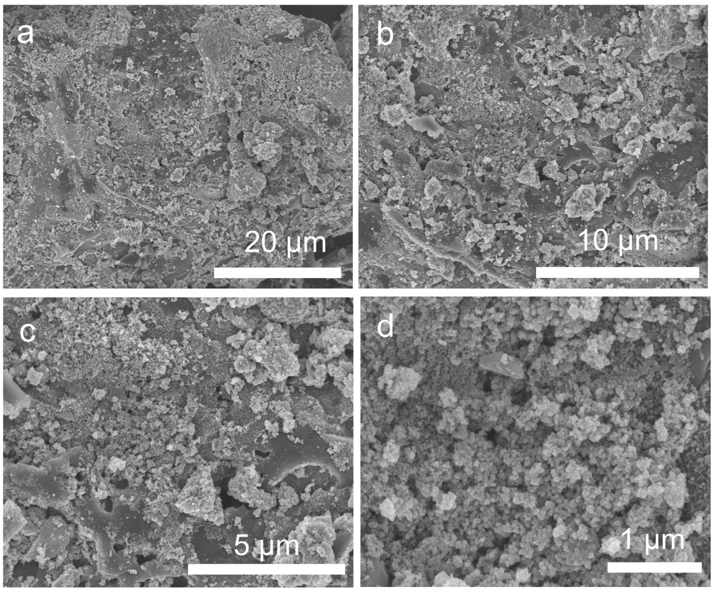

Figure S3. (a-d) SEM images of $CMC/Fe_3O_4/GBC$ at different magnifications.

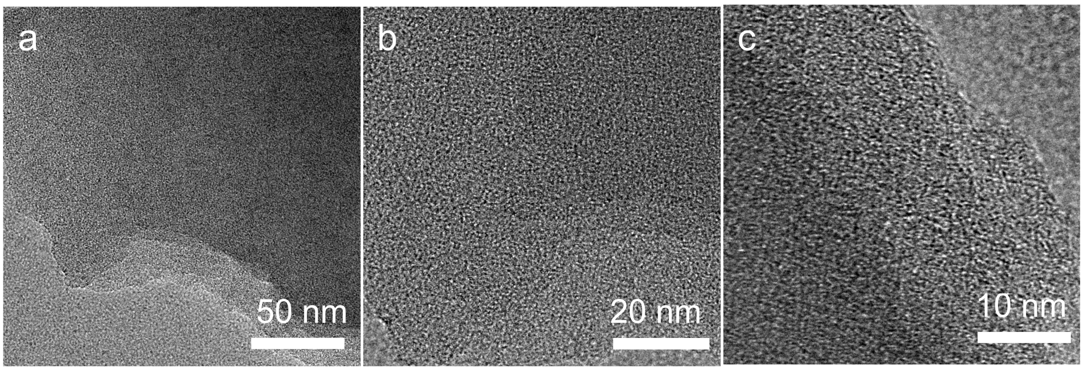

Figure S4. (a-c) TEM images of GBC at different magnifications.

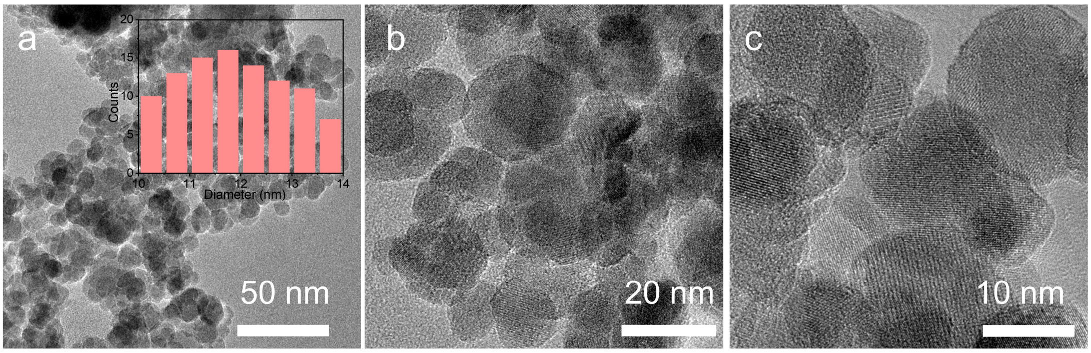

Figure S5. (a-c) TEM images of Fe_3O_4/GBC at different magnifications.

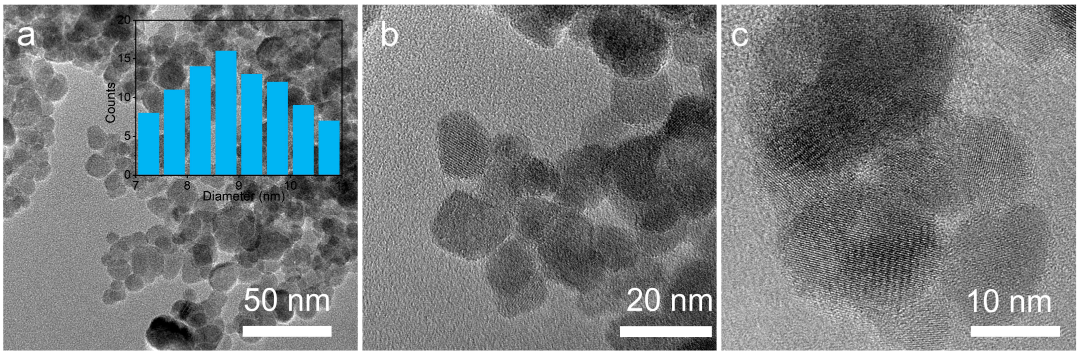

Figure S6. (a-c) TEM images of CMC/Fe_3O_4/GBC at different magnifications.

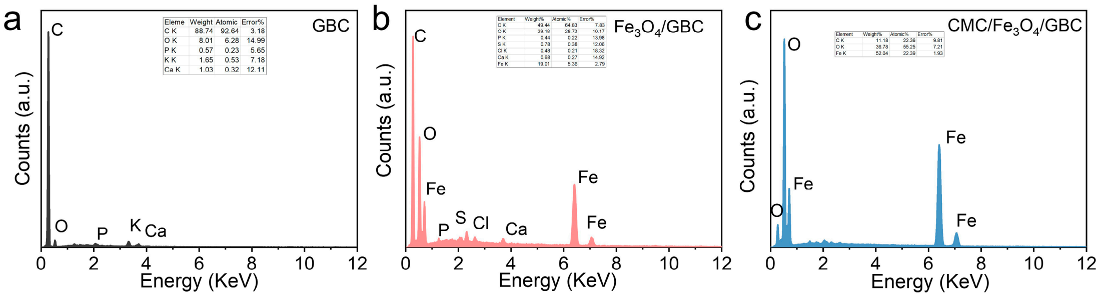

Figure S7. EDX spectra of (a) GBC, (b) Fe_3O_4/GBC, and (c) CMC/Fe_3O_4/GBC. Embedded tables in each inset summarize the atomic-percent elemental composition of each sample. Note that EDX cannot reliably quantify real trace-level impurities below ~1 at.%; reported impurity values represent detectable near-percent-level signals. EDX acquisition parameters: accelerating voltage = 20 kV; acquisition live-time = 120 s.

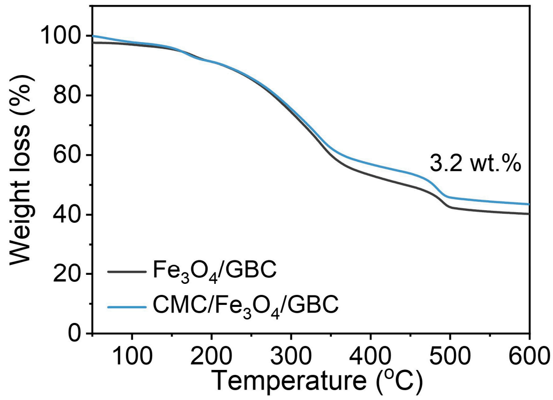

Figure S8. TG curves of Fe_3O_4/GBC and CMC/Fe_3O_4/GBC, respectively.

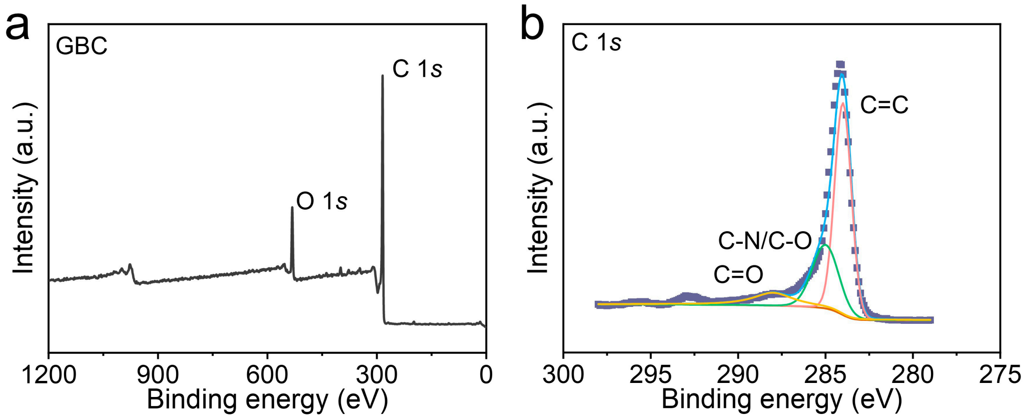

Figure S9. (a, b) Survey XPS spectrum and high-resolution C 1*s* spectrum of GBC, respectively.

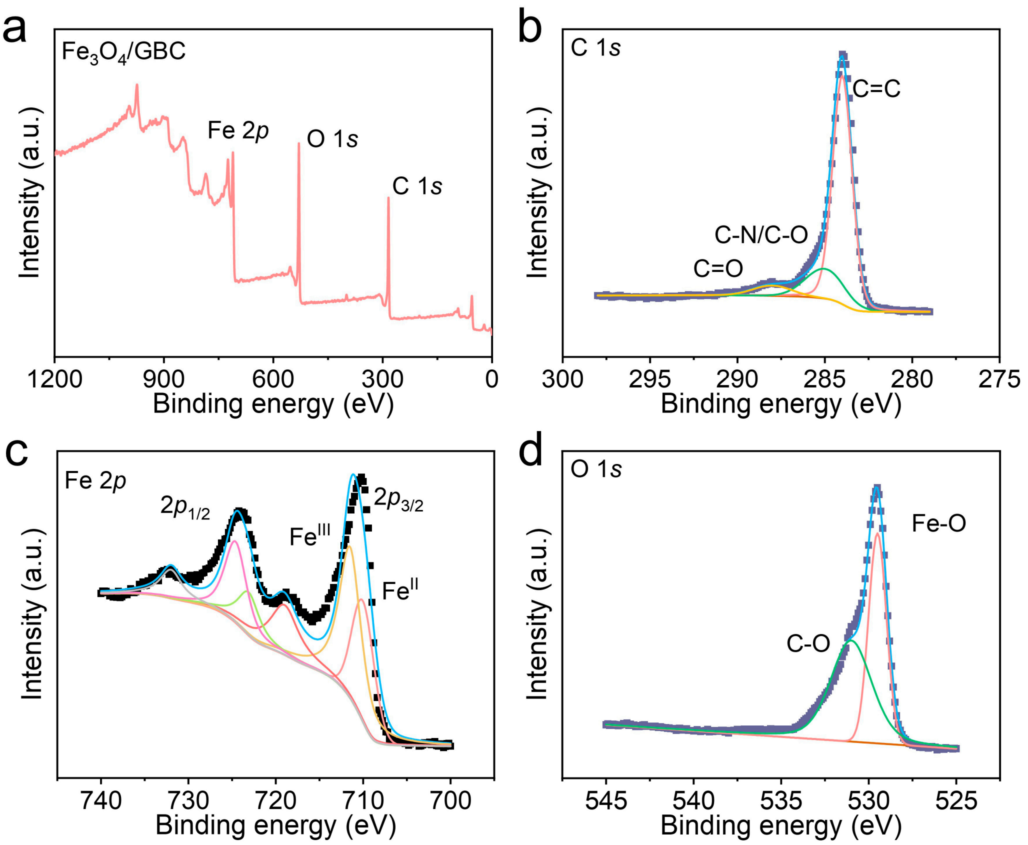

Figure S10. (a-d) Survey XPS spectrum and high-resolution C 1*s*, Fe 2*p*, and O 1*s* spectra of Fe_3O_4/GBC, respectively. Fe 2*p* spectrum fitted with additional shake-up satellite components at ~717 eV and ~727 eV for mixed-valence Fe_3O_4.

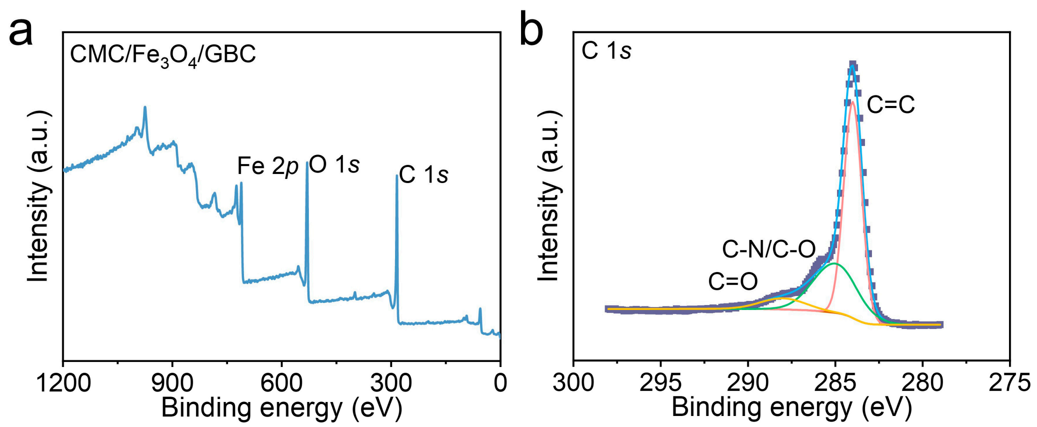

Figure S11. (a, b) Survey XPS spectrum and high-resolution C 1*s* spectrum of $CMC/Fe_3O_4/GBC$, respectively.

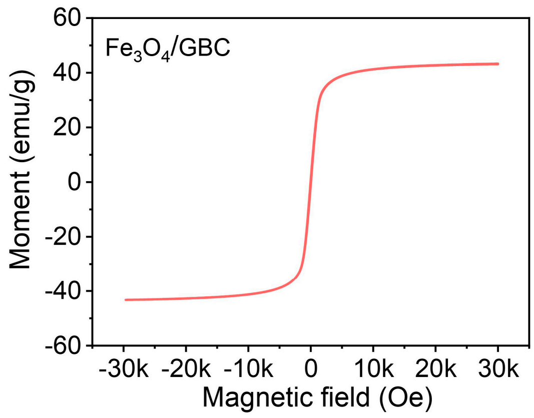

Figure S12. H-M curve of Fe_3O_4/GBC.

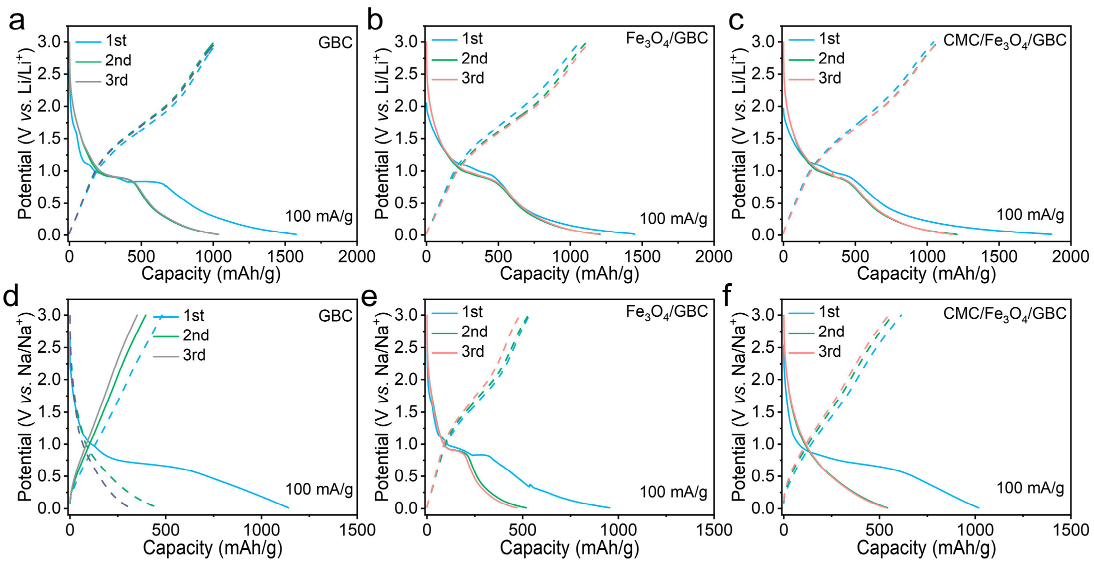

Figure S13. Discharge-charge curves of (a,d) GBC, (b,e) Fe_3O_4/GBC, and (c,f) $CMC/Fe_3O_4/GBC$ in the 1st, 2nd, and 3rd cycle, respectively.

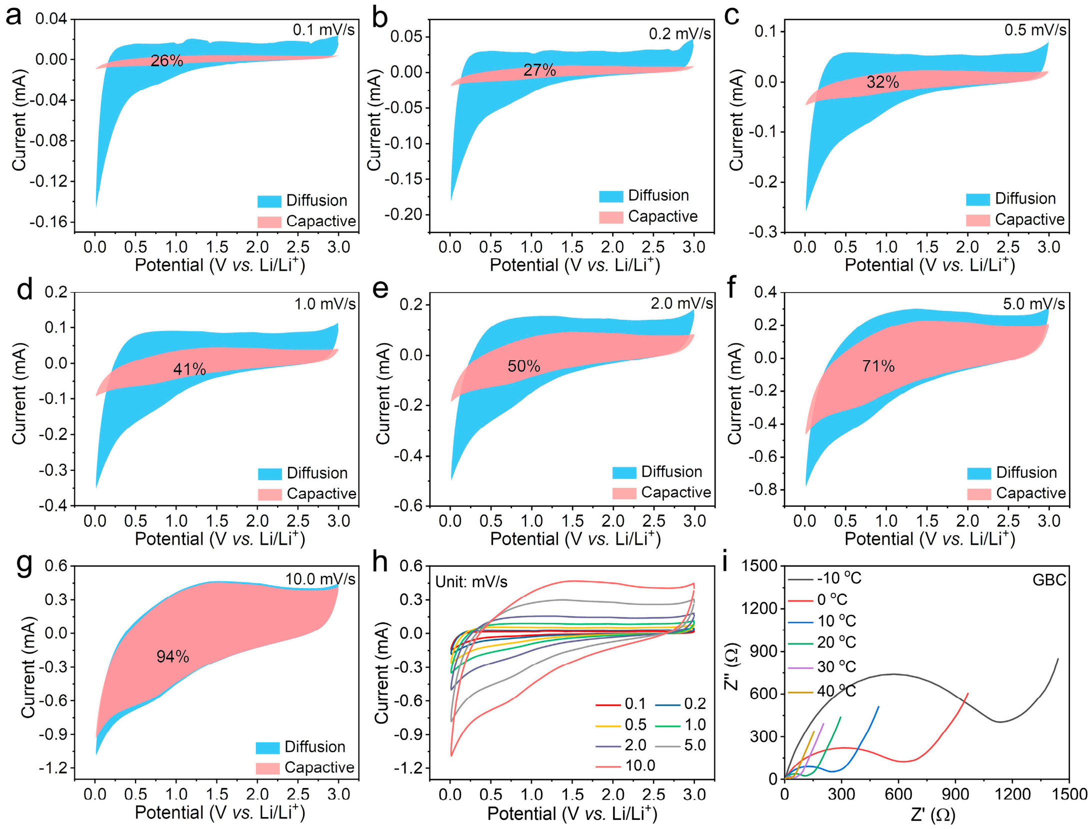

Figure S14. (a-g) CV curves and capacity contribution ratios of GBC in LIBs at various scan rates; (h) CV curves of GBC measured at different scan rates; (i) EIS curves collected at various temperatures.

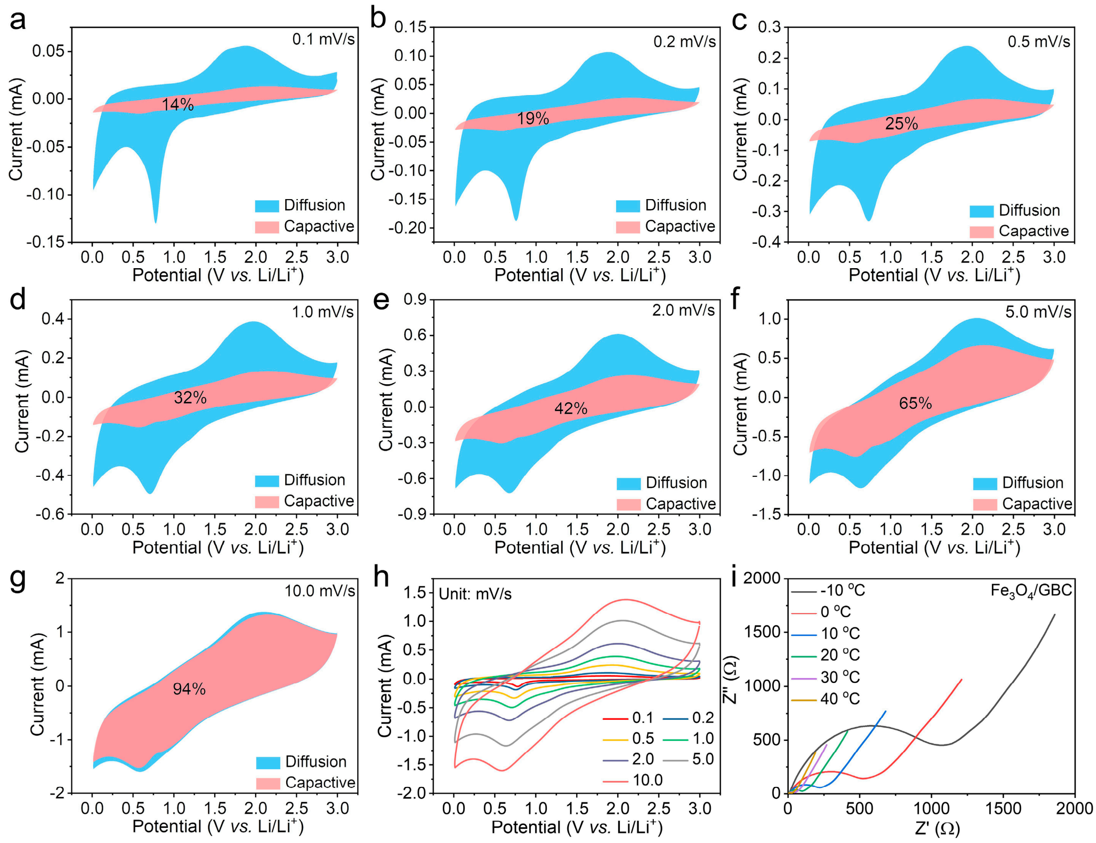

Figure S15. (a-g) CV curves and capacity contribution ratios of Fe_3O_4/GBC in LIBs at various scan rates; (h) CV curves of Fe_3O_4/GBC measured at different scan rates; (i) EIS curves collected at various temperatures.

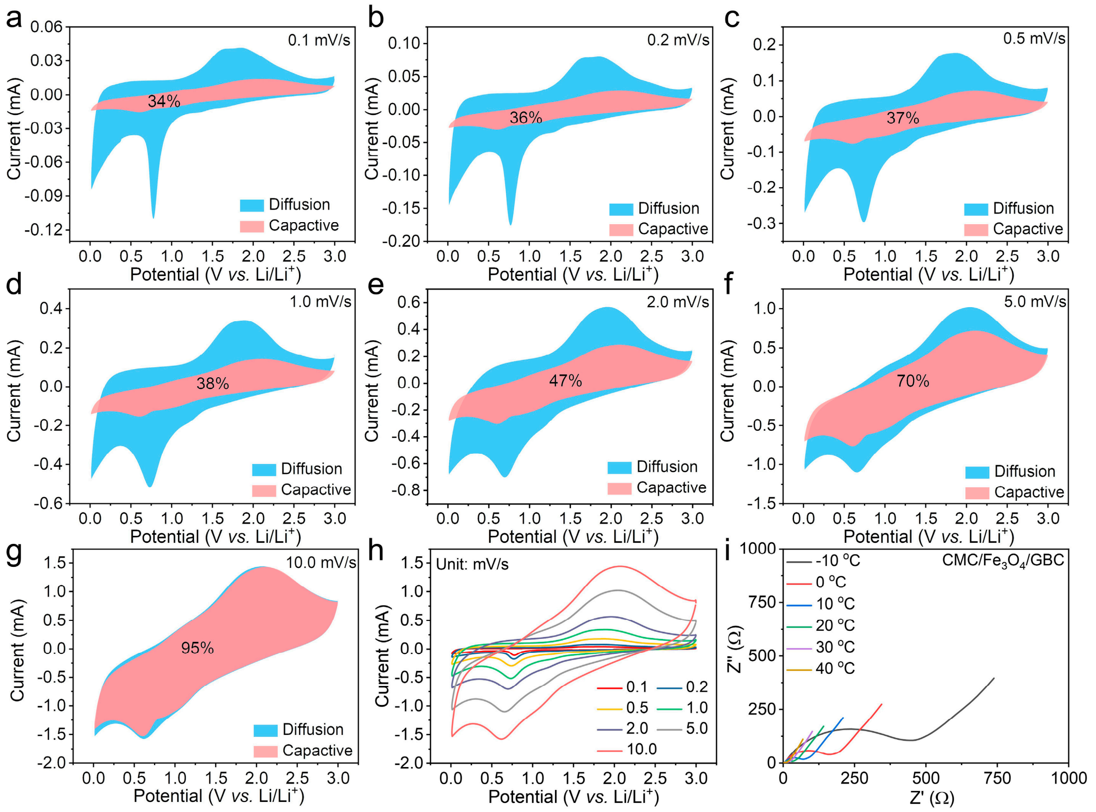

Figure S16. (a-g) CV curves and capacity contribution ratios of CMC/Fe_3O_4/GBC in LIBs at various scan rates; (h) CV curves of CMC/Fe_3O_4/GBC measured at different scan rates; (i) EIS curves collected at various temperatures.

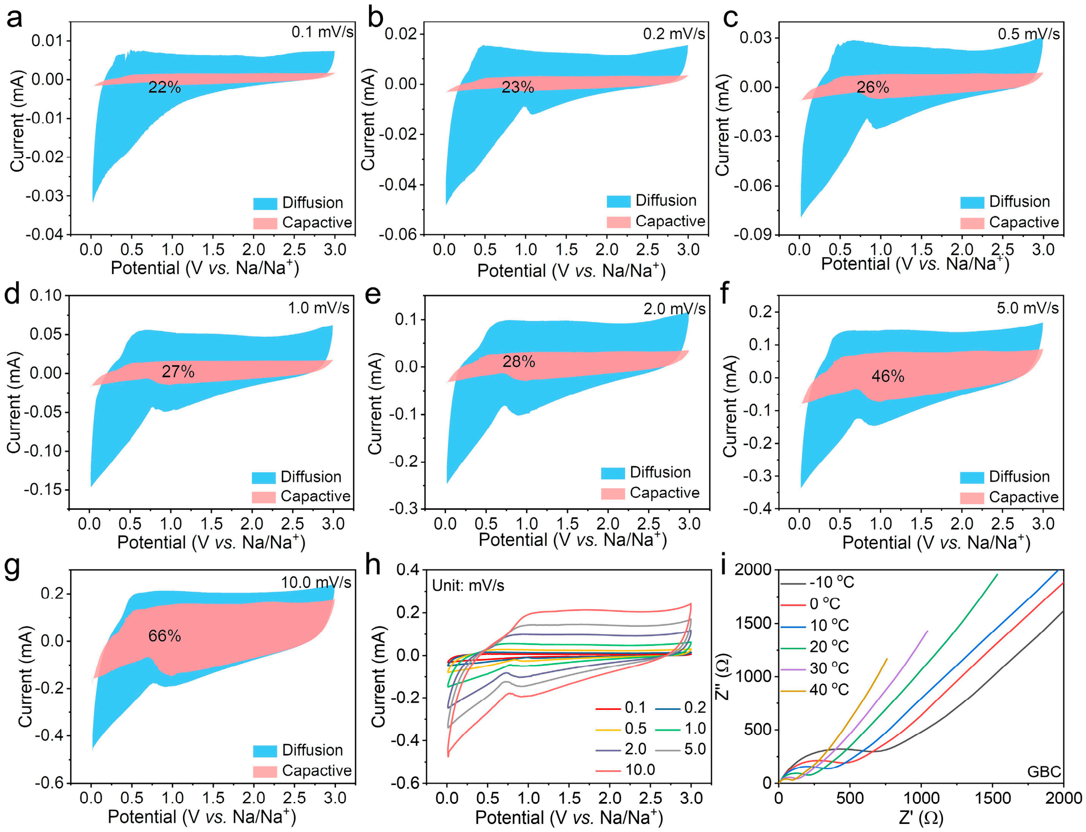

Figure S17. (a-g) CV curves and capacity contribution ratios of GBC in SIBs at various scan rates; (h) CV curves of GBC measured at different scan rates; (i) EIS curves collected at various temperatures.

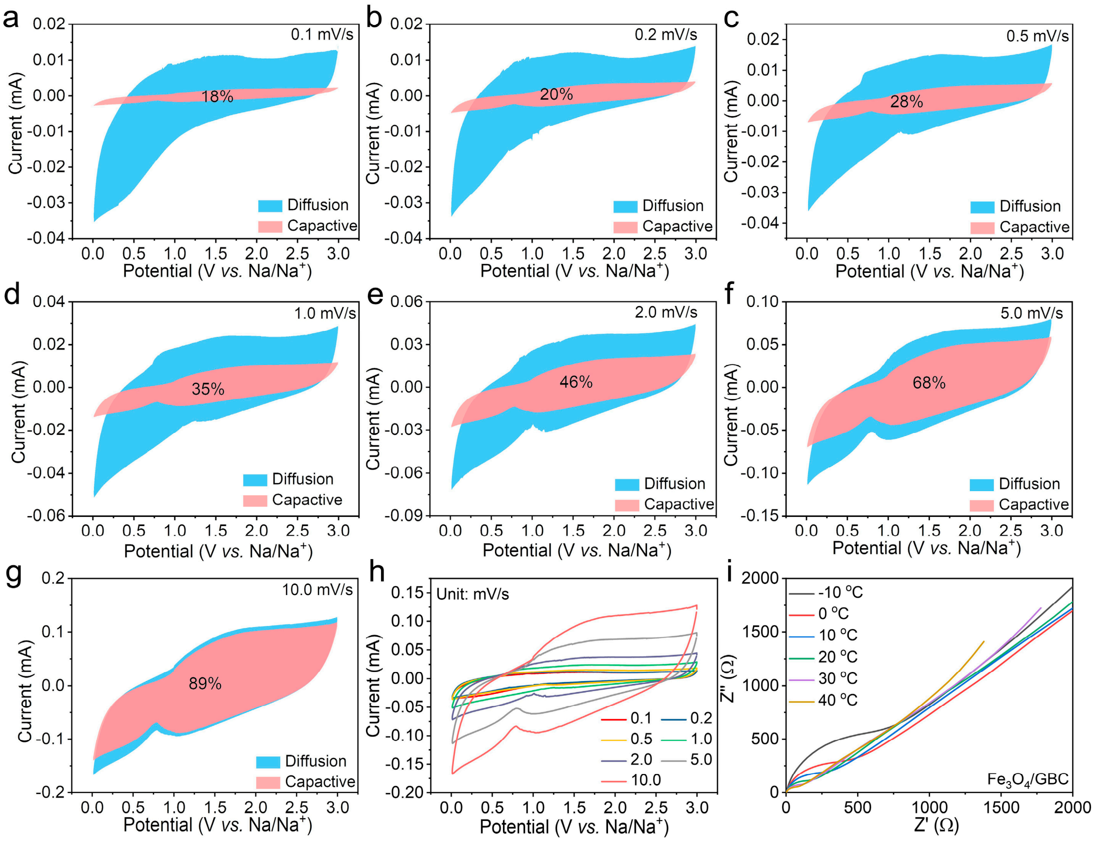

Figure S18. (a-g) CV voltammetry curves and capacity contribution ratios of Fe_3O_4/GBC in SIBs at various scan rates; (h) CV curves of Fe_3O_4/GBC measured at different scan rates; (i) EIS curves collected at various temperatures.

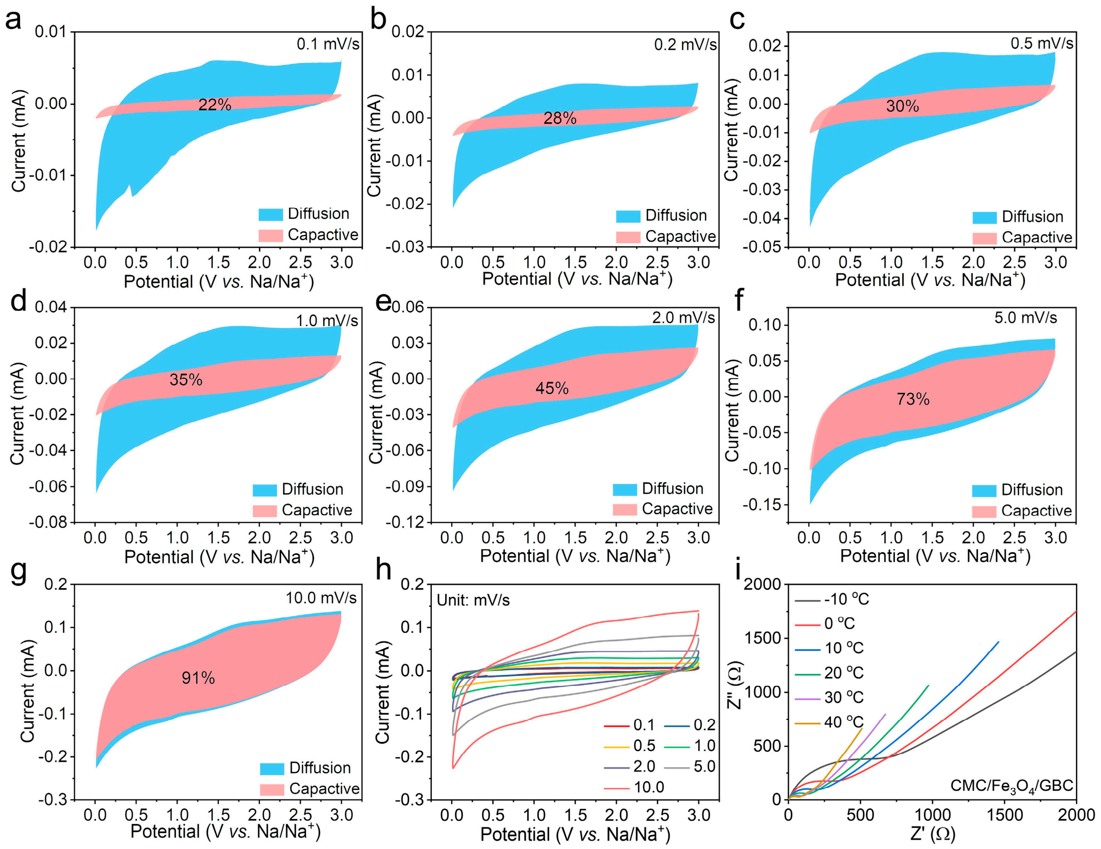

Figure S19. (a-g) CV curves and capacity contribution ratios of CMC/Fe_3O_4/GBC in SIBs at various scan rates; (h) CV curves of CMC/Fe_3O_4/GBC measured at different scan rates; (i) EIS curves collected at various temperatures.

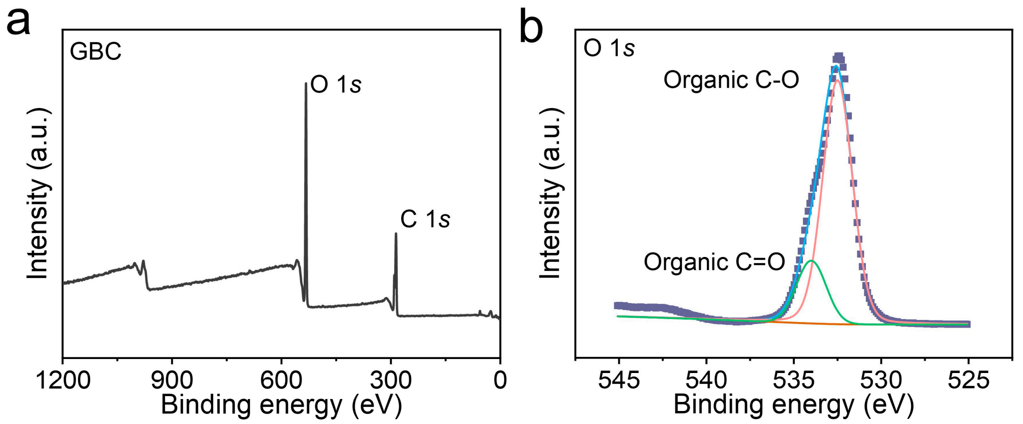

Figure S20. (a, b) Survey XPS spectrum and high-resolution O 1*s* spectrum of GBC after cycling in LIBs, respectively.

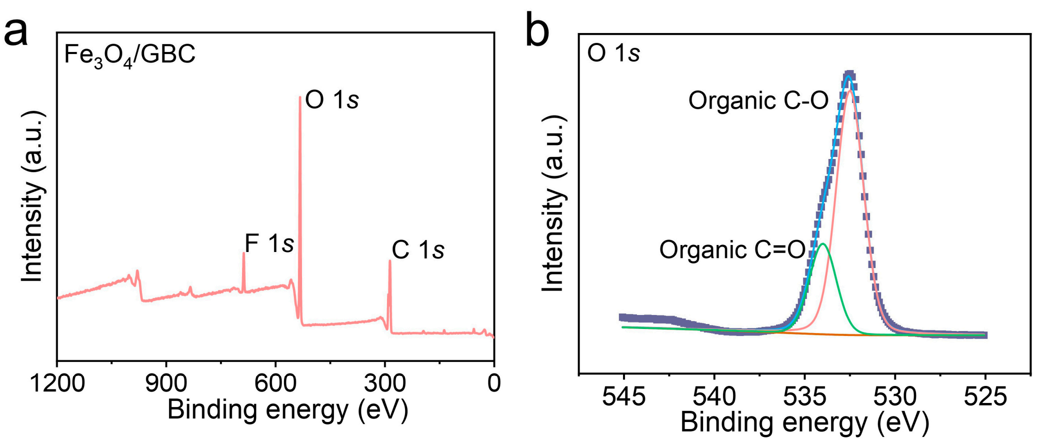

Figure S21. (a, b) Survey XPS spectrum and high-resolution O 1*s* spectrum of Fe_3O_4/GBC after cycling in LIBs, respectively.

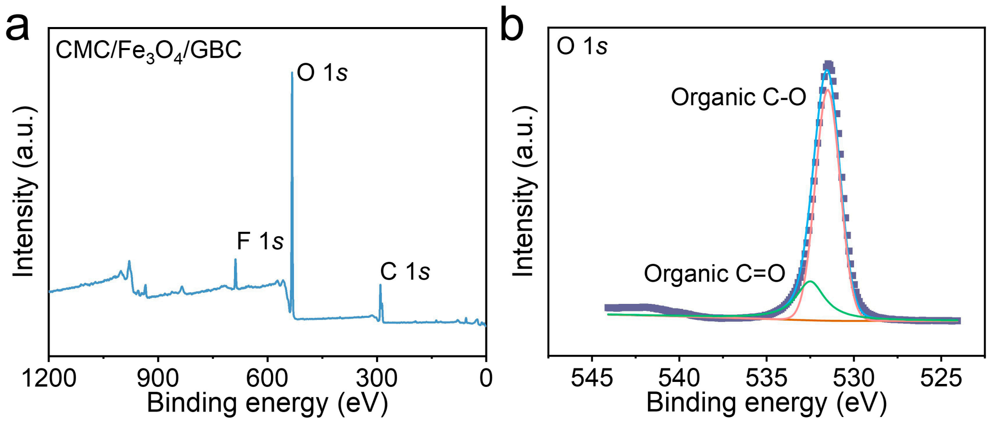

Figure S22. (a, b) Survey XPS spectrum and high-resolution O 1*s* spectrum of CMC/Fe_3O_4/GBC after cycling in LIBs, respectively.

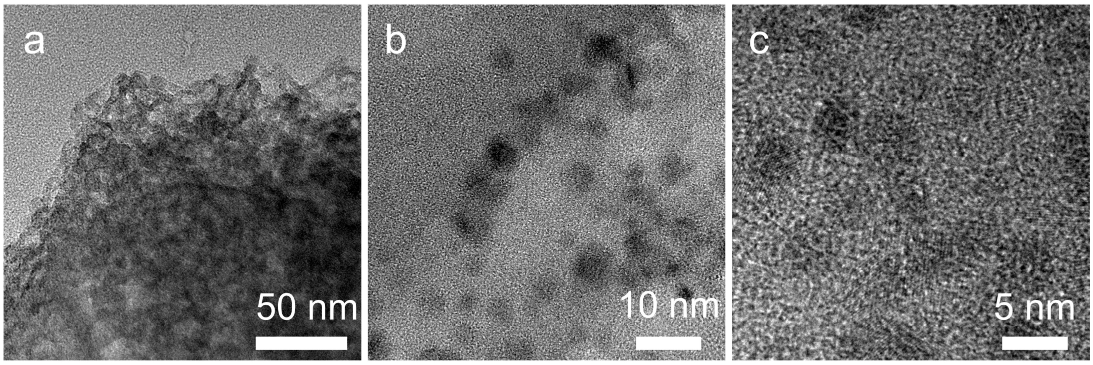

Figure S23. (a-c) TEM images of CMC/Fe_3O_4/GBC at different magnifications after cycling in LIBs, respectively.

Table S1. Comparison of the cyclic performance of various Fe_3O_4-based as anode materials for LIBs and SIBs at different current densities.

Sample	Current density	Cycle No.	Capacity mAh/g	Ref.
CMC/Fe_3O_4/GBC	2000 mA/g	2000	@LIB<br>@SIB	**This work**
Fe_3O_4	100 mA/g	100	556@LIB	Int. J. Electrochem. Sci., 2022, 17, 220663
Fe_3O_4@N/C	1000 mA/g<br>5000 mA/g	350<br>250	1200@LIB<br>237@SIB	New J. Chem., 2022, 46, 10229
Fe_3O_4/rGO	100 mA/g	1000	1224@LIB	Carbon, 2024, 222, 119003
N,S co-doped Fe_3O_4/C	1000 mA/g	1000	925@LIB	Nano Mater. Sci., 2026, 8,641
Fe_3O_4@CNTs	1000 mA/g	100	683@LIB	App. Sur. Sci., 2024, 653, 159333
Fe_3O_4/C	100 mA/g	35	802@LIB	J. Mater. Sci., 2024, 35, 1816
Fe_3O_4@rGO	100 mA/g	100	342@SIB	Chem. Commun., 2025, 61, 15007
Fe_3O_4/Fe@CN	2000 mA/g	500	244@SIB	Small 2025, 21, e07287
Combusted Fe_3O_4 NP	54 mA/g	100	100@SIB	ACS Appl. Mater. Interfaces 2022, 14, 50773
Fe_3O_4/FeS_2/C	2000 mA/cm^2	1000	0.5@LIB	Chem. Eng. J., 2023, 455, 140945
Fe_3O_4-CNF	500 mA/g	200	358@SIB	J. Alloys Comp., 2022, 918, 165672
Fe_3O_4@C@G	1000 mA/g<br>100 mA/g	1000<br>600	400@LIB<br>180@SIB	J. Coll. Inter. Sci., 2020, 565, 494
Fe_3O_4/Fe_3Se_4	1000 mA/g	500	270@SIB	Chem. Eng. Sci., 2026, 335, 124457
